# Supplementary material for: Fine-scale spatial and social patterns of SARS-CoV-2 transmission from identical pathogen sequences
Source: medRxiv. 2024 May 25:2024.05.24.24307811. Preprint. [Version 1] doi: 10.1101/2024.05.24.24307811 (PMC11142302; doi:10.1101/2024.05.24.24307811)
Supplement: Supplementary file 1 [file NIHPP2024.05.24.24307811V1-supplement-1.pdf]

medRxiv preprint doi: https://doi.org/10.1101/2024.05.24.24307811; this version posted May 25, 2024. The copyright holder for this preprint
(which was not certified by peer review) is the author/funder, who has granted medRxiv a license to display the preprint in perpetuity.
This article is a US Government work. It is not subject to copyright under 17 USC 105 and is also made available
for use under a CC0 license.

Supplementary materials for:

Fine-scale spatial and social patterns of SARS-CoV-2 transmission from identical pathogen sequences

List of supplementary figures

S1 Permutation test to explore the spatio-temporal signal in clusters of identical SARS-CoV-2 sequences. . . . 31
S2 The magnitude of the relative risk of observing sequences at a given genetic distance within the same county is impacted by transmission intensity. 32
S3 Our measure of relative risk corrects for uneven sequencing between regions. . . . 33
S4 Simulation study exploring the impact of sequencing bias on results from a discrete trait analysis and from our RR framework. . . . 34
S5 Relative risk of observing identical sequences in two counties. . . . 35
S6 Relative risk of observing pairs of identical sequences between counties. . . . 37
S7 Impact of subsampling on the significance of the association between the relative risk of identical sequences and whether counties and ZCTAs are adjacent or not. 38
S8 Distribution of the delay between sequence collection within pairs of identical sequences collected between Eastern and Western WA and across epidemic waves. 39
S9 Comparison between the number of directed commuting flows and the number of directed visits between two counties. . . . 39
S10 Comparison between the relative risk of observing identical sequences and the relative risk of movement at the county level. . . . 40
S11 Geographical regions used to aggregate counties. . . . 41
S12 Comparison between the relative risk of observing identical sequences and the relative risk of movement at the region level. . . . 42
S13 Relationship between the relative risk of observing identical sequences in two regions and the relative risk of movement between these regionsobtained from mobile phone mobility data across epidemic waves 43
S14 Comparison of the total number of visits between pairs of WA counties across the 4 epidemic waves during our study period. . . . 44
S15 Comparison of connectivity metrics across the Eastern / Western WA border among counties located on the border. . . . 45
S16 Patterns of occurrence of pairs of identical sequences between ZCTAs in Pierce and Mason counties, the two counties that are home of WA female prisons. . . . 46
S17 Relative risk for pairs of identical sequences of being observed between two age groups. . . . 47
S18 Relative risk for pairs sequences of being observed between two age groups depending on their genetic distance. . . . 48
S19 Impact of the spatial scale on the relative risk for pairs sequences of being observed between two age groups. . . . 49
S20 Median delay between the dates of sequence collection within pairs of identical sequences 50
S21 Sensitivity analysis on the timing of pairs identical sequences between age groups using symptom onset dates 51

medRxiv preprint doi: https://doi.org/10.1101/2024.05.24.24307811; this version posted May 25, 2024. The copyright holder for this preprint (which was not certified by peer review) is the author/funder, who has granted medRxiv a license to display the preprint in perpetuity.
This article is a US Government work. It is not subject to copyright under 17 USC 105 and is also made available for use under a CC0 license.

S22 Ratio between the relative risk of observing identical sequences within a given vaccination group (denoted V_1) and between two vaccination groups (denoted V_1 and V_2). . . . 51
S23 Impact of non-sampled locations on the computation of the RR. . . . 52
S24 Impact of dataset size on the number of clusters of identical sequences and the number of sequences with another identical sequence in the dataset. . . . 52
S25 Impact of the number of groups included in the analysis on the dataset size required for the error relative risk of observing identical sequences to be lower than 10%. . . . 53
S26 Empirical distribution of the delay between symptom onset and sequence collection by age (rows), period (columns) and geographic region (colours). . . . 54
S27 Time-series of COVID-19 cases in WA over the study periods. . . . 55
S28 Illustration of the downsampling strategy used to quantify the amount of data required to compute relative risks. . . . 55

List of supplementary tables

S1 Performance of Discrete Trait Analysis (DTA) and our relative risk metric (RR) in quantifying migration patterns. . . . 56
S2 Comparison of the relative of risk of observing identical sequences at the ZCTA level by adjacency level. . . . 56
S3 Comparison between the relative risk of observing identical sequences between two geographic regions and the risk of movement between different geographies. . . 57
S4 Characteristics of WA male prisons. . . . 57

medRxiv preprint doi: https://doi.org/10.1101/2024.05.24.24307811; this version posted May 25, 2024. The copyright holder for this preprint
(which was not certified by peer review) is the author/funder, who has granted medRxiv a license to display the preprint in perpetuity.
This article is a US Government work. It is not subject to copyright under 17 USC 105 and is also made available
for use under a CC0 license.

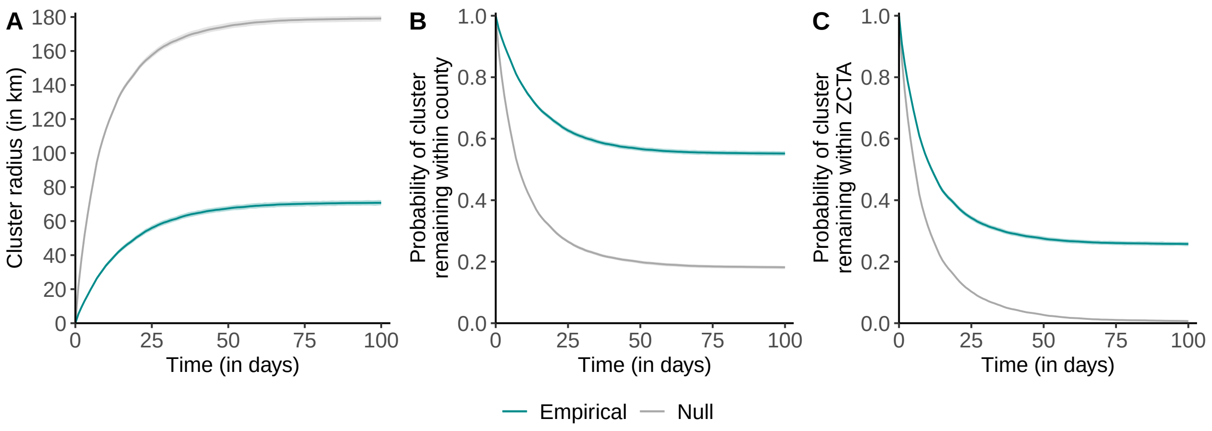

Figure S1. Permutation test to explore the spatio-temporal signal in clusters of identical SARS-CoV-2 sequences. A. Radius of clusters of identical sequences as a function of time since first sequence collection. **B.** Probability for all sequences within a cluster of identical sequences of remaining in the same county as a function of time since first sequence collection. **C.** Probability for all sequences within a cluster of identical sequences of remaining in the same ZCTA as a function of time since first sequence collection. The grey shaded areas correspond to 95% confidence intervals of a null distribution generated from 100 simulation where the geographical location of sequences from WA Sentinel surveillance are permuted. The grey lines correspond to the medians the simulated null distributions.

medRxiv preprint doi: https://doi.org/10.1101/2024.05.24.24307811; this version posted May 25, 2024. The copyright holder for this preprint
(which was not certified by peer review) is the author/funder, who has granted medRxiv a license to display the preprint in perpetuity.
This article is a US Government work. It is not subject to copyright under 17 USC 105 and is also made available
for use under a CC0 license.

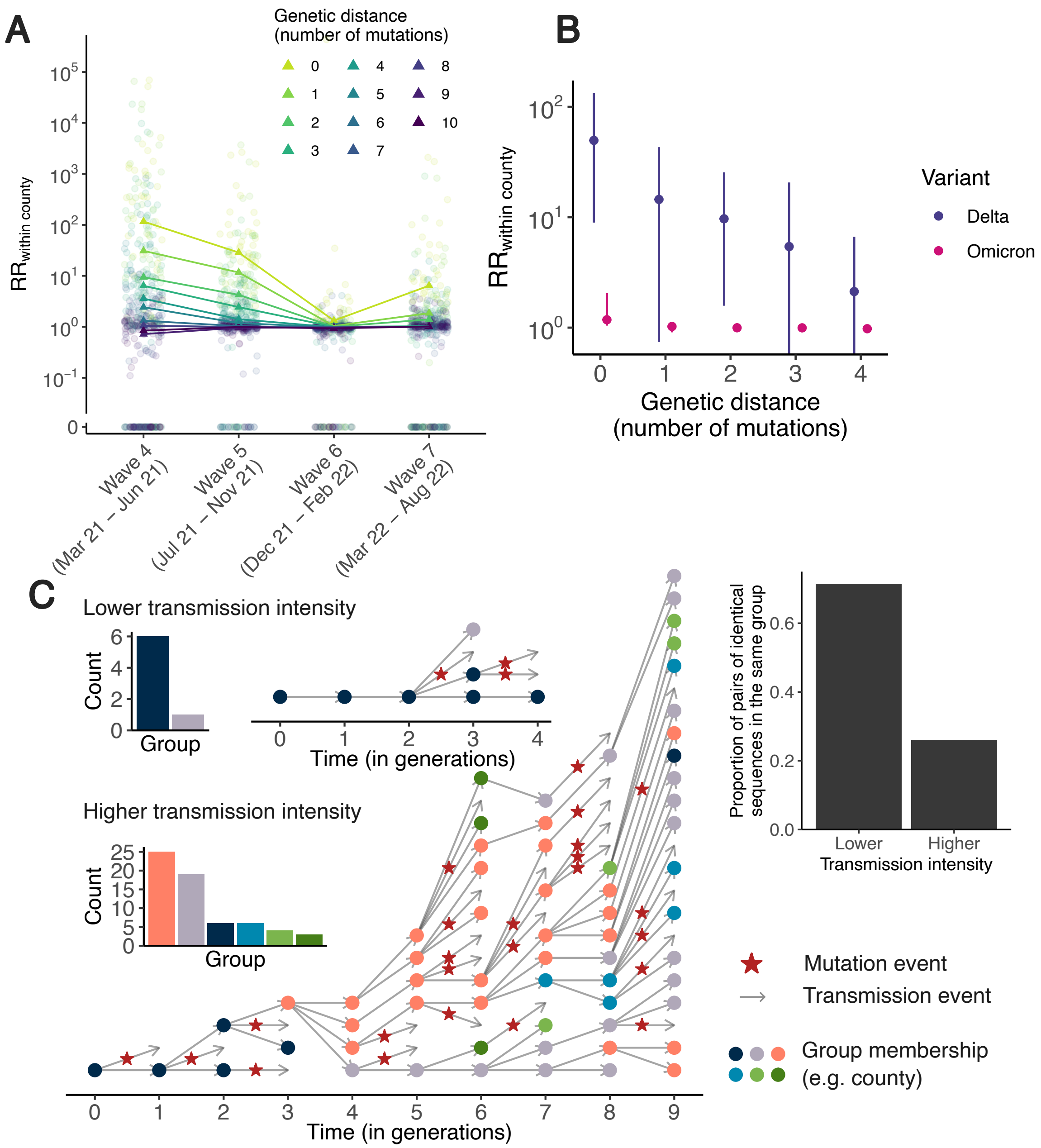

Figure S2. The magnitude of the relative risk of observing sequences at a given genetic distance within the same county is impacted by transmission intensity. A. Relative risk of observing sequences at a given genetic distance within the same county across multiple epidemic waves. We defined waves as: March 2021-June 2021 (Wave 4), July 2021-November 2021 (Wave 5), December 2021-February 2022 (Wave 6) and March 2022-August 2022 (Wave 7). In A, circular points correspond to individuals counties and triangles correspond to the median across counties. **B.** Median relative risk of observing pairs sequences within the same county (with IQR) as a function of genetic distance stratified by variant during Wave 6. **C.** A higher transmission intensity results in larger clusters of identical sequences that tend to be more mixed across groups. In C, the two clusters are simulated using a branching process with mutation [6] by assuming the probability for an infector and an infectee to have the same consensus sequence equal to 0.69 and a probability for an infectee of being in the same groups as its infector of 0.7. We consider a reproduction number of 1.2 for the lower transmission intensity scenario and of 2.0 in the higher transmission intensity scenario.

medRxiv preprint doi: https://doi.org/10.1101/2024.05.24.24307811; this version posted May 25, 2024. The copyright holder for this preprint (which was not certified by peer review) is the author/funder, who has granted medRxiv a license to display the preprint in perpetuity.
This article is a US Government work. It is not subject to copyright under 17 USC 105 and is also made available for use under a CC0 license.

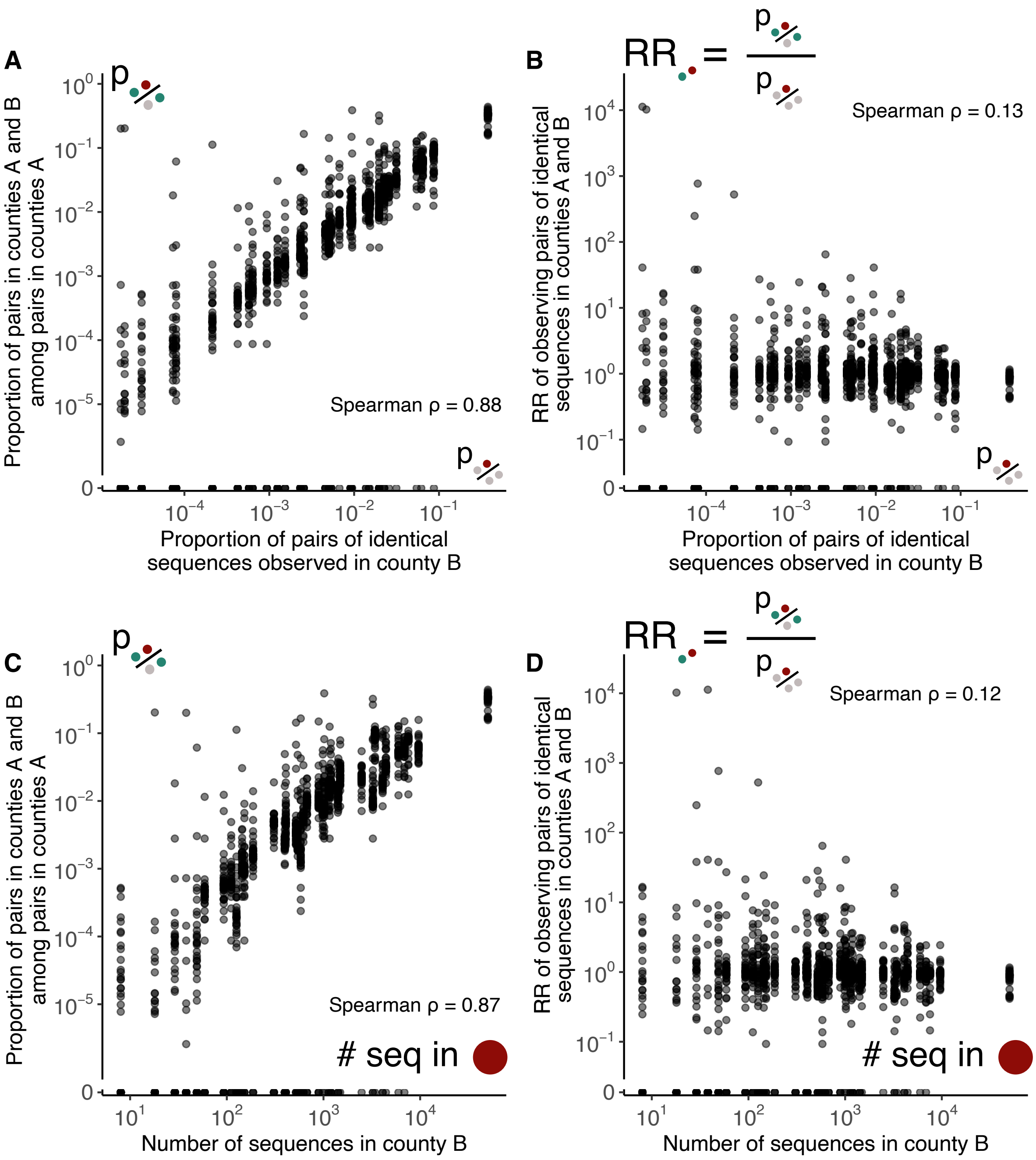

Figure S3. Our measure of relative risk corrects for uneven sequencing between regions. **A.** Proportion of pairs of identical sequences shared between counties A and B among pairs observed in county A as a function of the proportion of pairs of identical sequences observed in county B. **B.** Relative risk for pairs of identical sequences of being observed in counties A and B as a function of the proportion of pairs of identical sequences observed in county B. **C.** Proportion of pairs of identical sequences shared between counties A and B as a function of the number of sequences available in county B. **D.** Relative risk for pairs of identical sequences of being observed in counties A and B as a function of the number of sequences available in county B.

medRxiv preprint doi: https://doi.org/10.1101/2024.05.24.24307811; this version posted May 25, 2024. The copyright holder for this preprint
(which was not certified by peer review) is the author/funder, who has granted medRxiv a license to display the preprint in perpetuity.
This article is a US Government work. It is not subject to copyright under 17 USC 105 and is also made available
for use under a CC0 license.

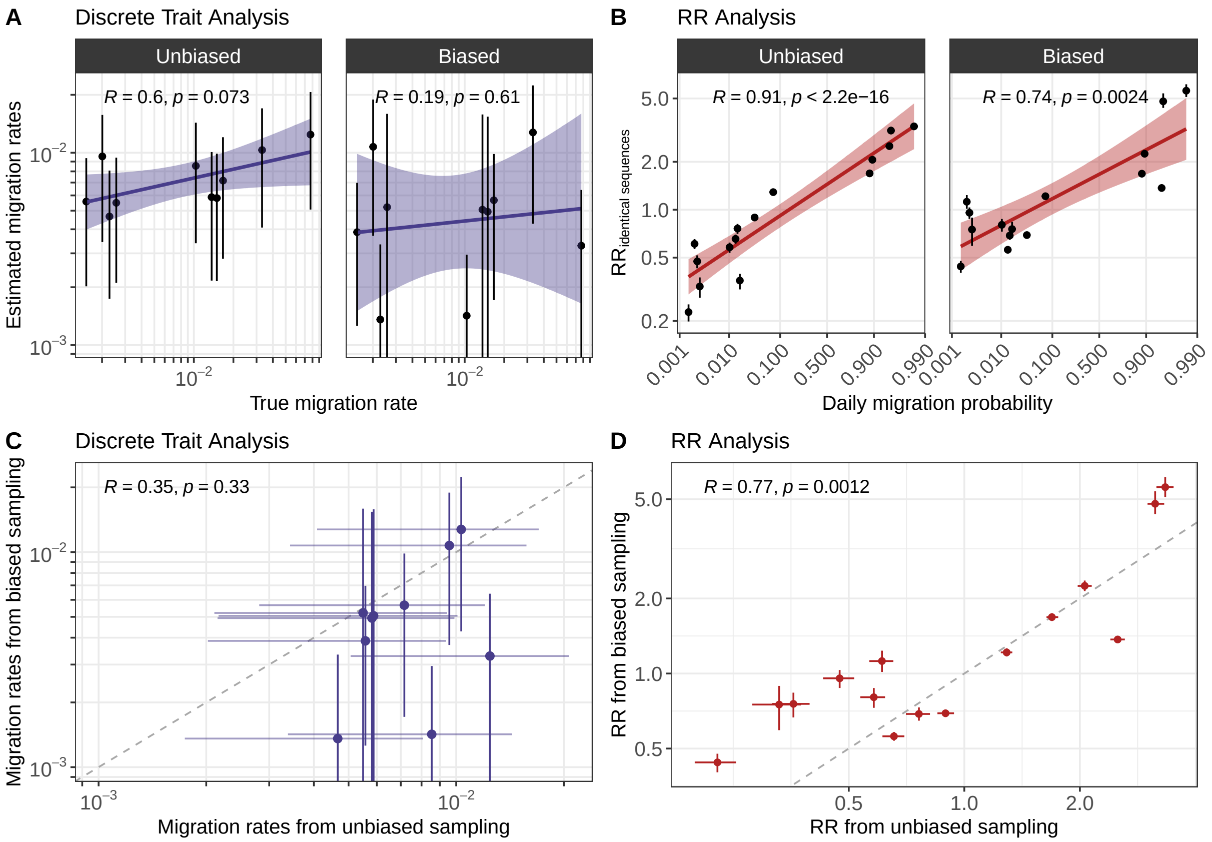

Figure S4. Simulation study exploring the impact of sequencing bias on results from a discrete trait analysis and from our RR framework. **A.** Comparison between migration rates estimated from a discrete trait analysis and the true migration rates used to simulate the sequence data. **B.** Comparison between the relative risk of observing identical sequences between two demes and the weekly migration probability between demes. **C.** Comparison between migration rates inferred from a sequence dataset generated in a biased sampling and an unbiased sampling scenario. **D.** Comparison between the relative risk of observing identical sequences in two groups from a sequence dataset generated in a biased sampling and an unbiased sampling scenario. For the RR, segments indicate 95% subsampling confidence intervals. For the migration rates, segments indicate 95% highest posterior density intervals. For each plot, we indicate the Spearman correlation coefficient (and the associated p-value).

medRxiv preprint doi: https://doi.org/10.1101/2024.05.24.24307811; this version posted May 25, 2024. The copyright holder for this preprint
(which was not certified by peer review) is the author/funder, who has granted medRxiv a license to display the preprint in perpetuity.
This article is a US Government work. It is not subject to copyright under 17 USC 105 and is also made available
for use under a CC0 license.

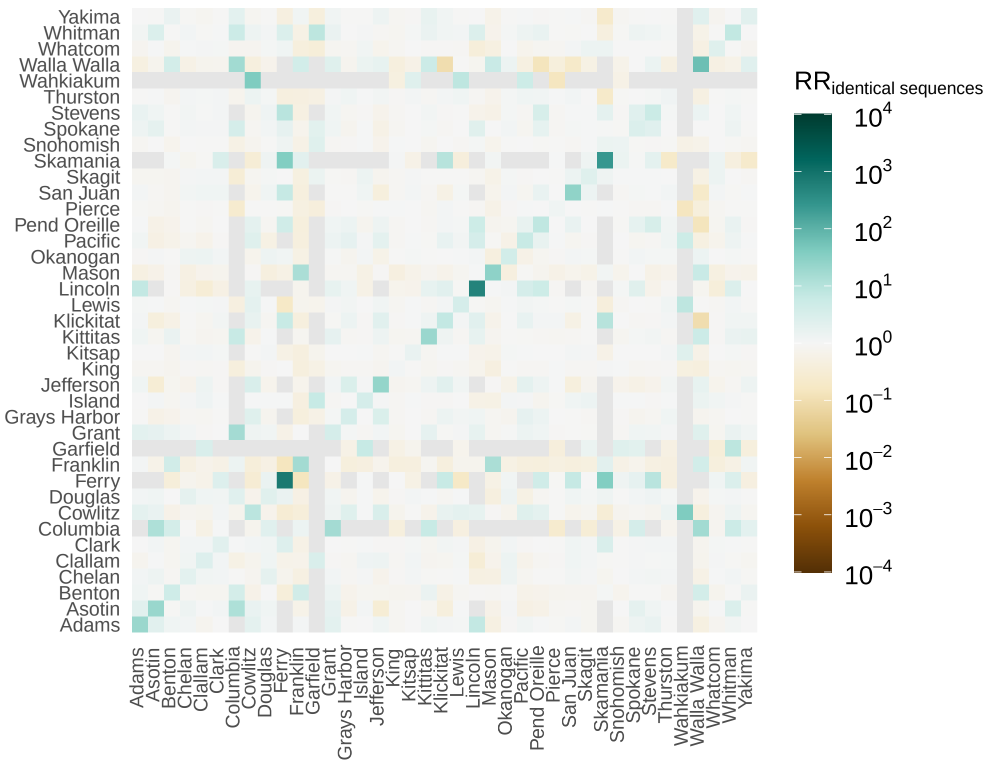

Figure S5. Relative risk of observing identical sequences in two counties. Grey squares correspond to pairs of counties between which no pairs of identical sequences were observed during the study period.

medRxiv preprint doi: https://doi.org/10.1101/2024.05.24.24307811; this version posted May 25, 2024. The copyright holder for this preprint (which was not certified by peer review) is the author/funder, who has granted medRxiv a license to display the preprint in perpetuity.
This article is a US Government work. It is not subject to copyright under 17 USC 105 and is also made available for use under a CC0 license.

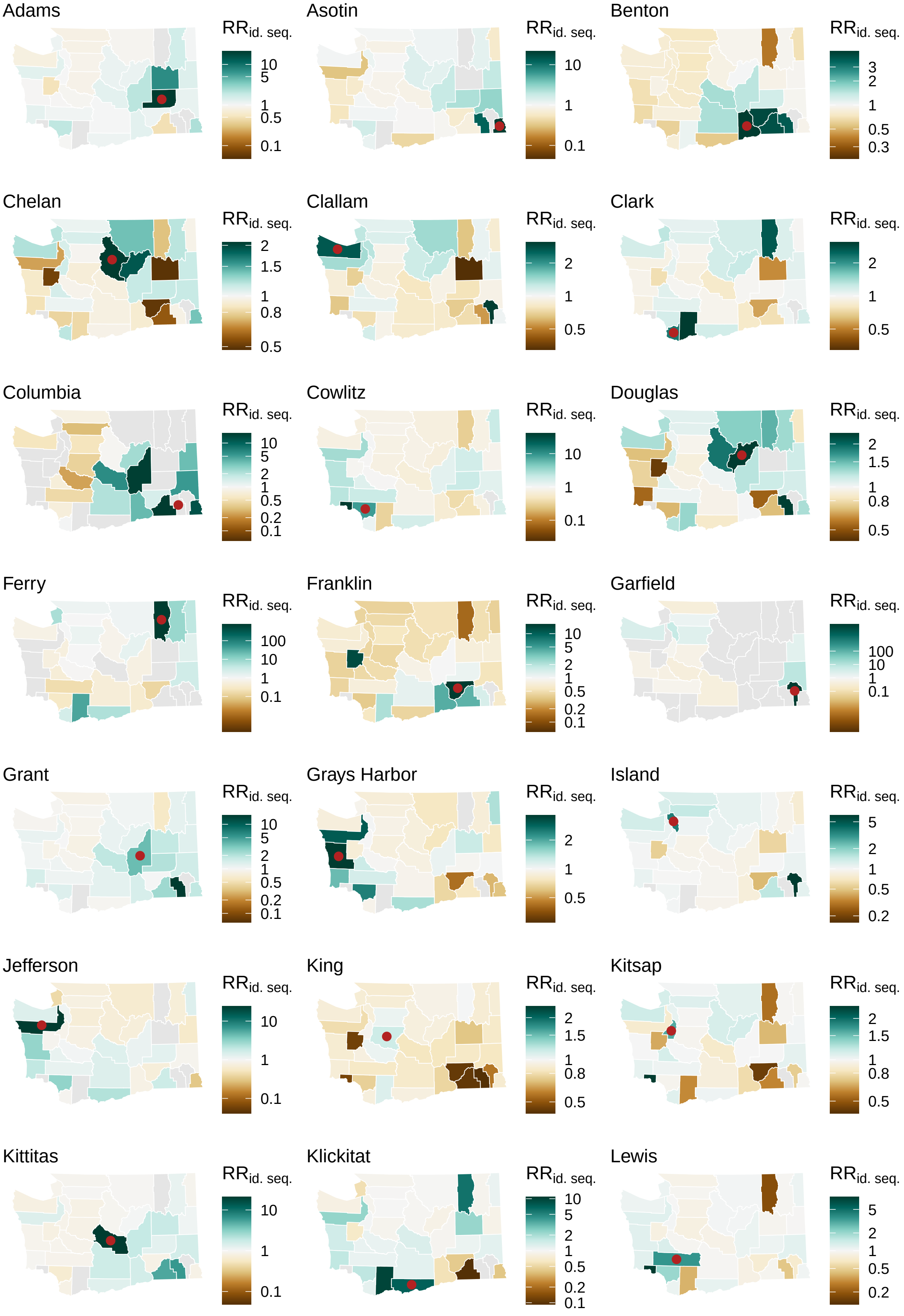

medRxiv preprint doi: https://doi.org/10.1101/2024.05.24.24307811; this version posted May 25, 2024. The copyright holder for this preprint (which was not certified by peer review) is the author/funder, who has granted medRxiv a license to display the preprint in perpetuity.
This article is a US Government work. It is not subject to copyright under 17 USC 105 and is also made available for use under a CC0 license.

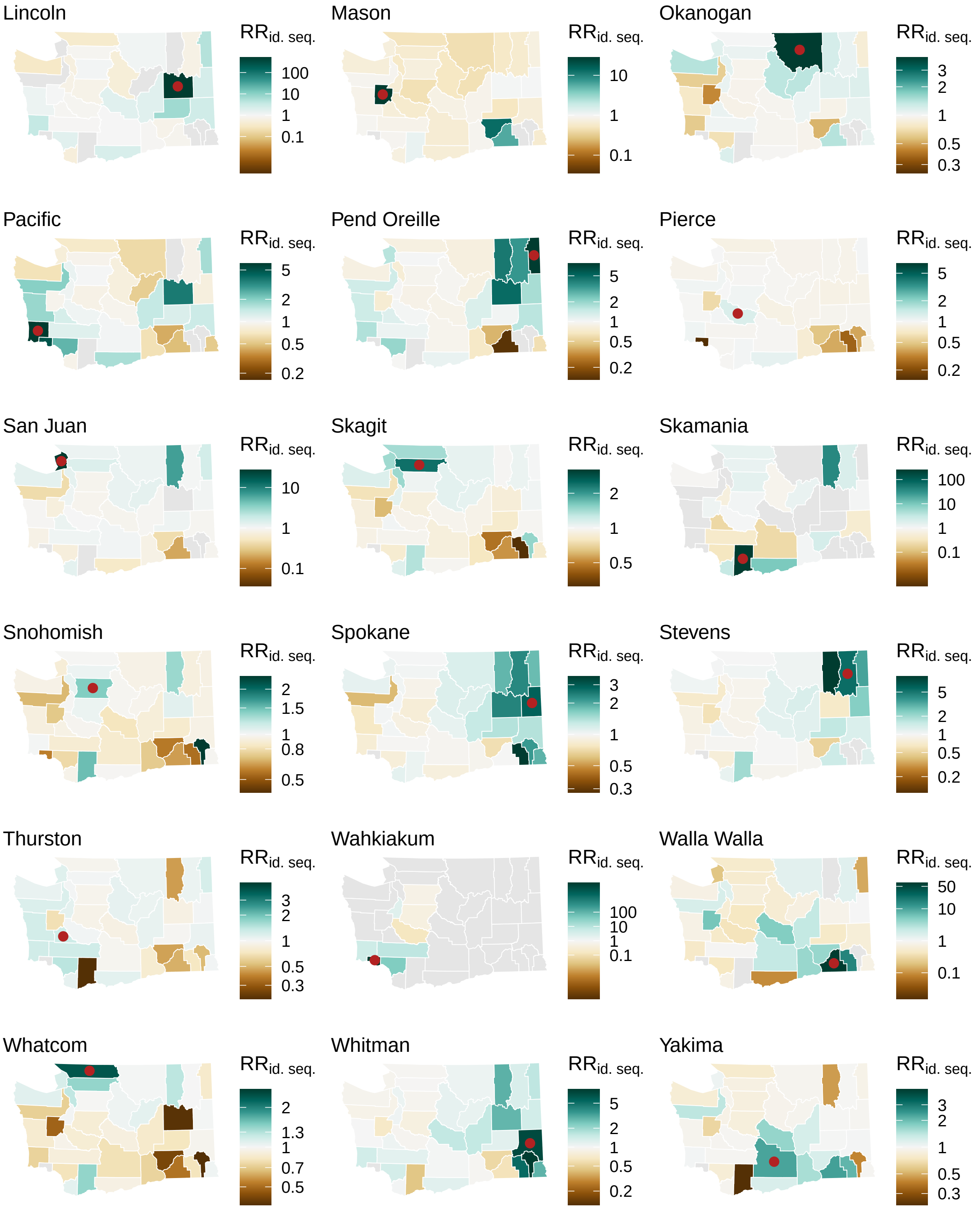

Figure S6. Relative risk of observing pairs of identical sequences between counties. On each map, we represent the relative risk of observing pairs of identical sequences in the county indicated by a red point (map title) and all the other counties in Washington state. Areas are colored in grey when no pairs of identical sequences are observed. To increase readability, each map has its own color scale.

medRxiv preprint doi: https://doi.org/10.1101/2024.05.24.24307811; this version posted May 25, 2024. The copyright holder for this preprint (which was not certified by peer review) is the author/funder, who has granted medRxiv a license to display the preprint in perpetuity.
This article is a US Government work. It is not subject to copyright under 17 USC 105 and is also made available for use under a CC0 license.

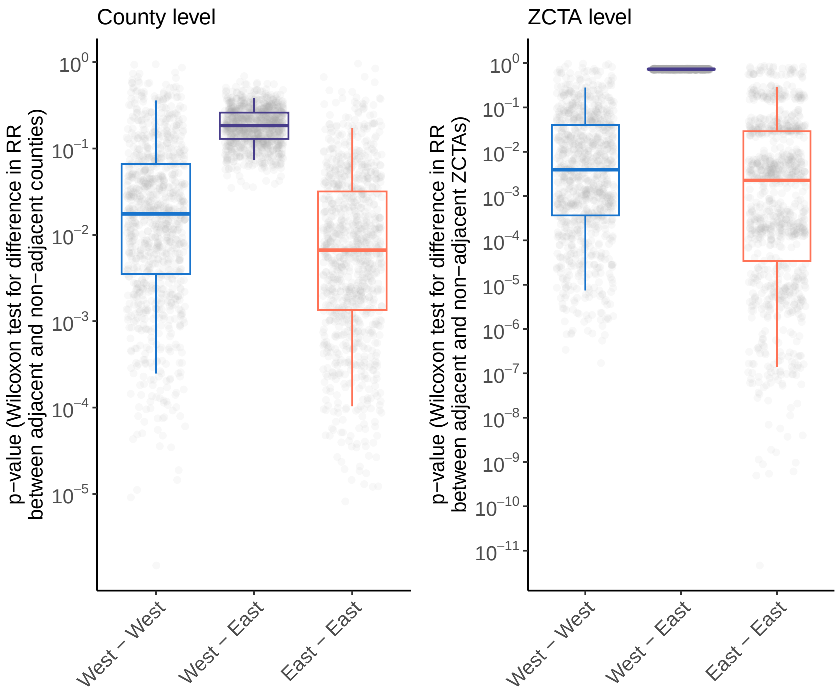

Figure S7. Impact of subsampling on the significance of the association between the relative risk of identical sequences and whether counties and ZCTAs are adjacent or not. We investigate whether our conclusions regarding the significance of the association between the relative risk of identical sequences falling in two distinct counties / ZCTAs and their adjacency status (adjacent / non-adjacent) can be impacted by the number of pairs of counties involved in the comparison (within Eastern WA, within Western WA and between Eastern and Western WA). At the county level, we subsample the pairs of counties involved in these 3 comparisons to 12 adjacent pairs of counties (number of pairs of adjacent counties between Eastern and Western WA) and 132 non-adjacent pairs of counties (number of pairs of non-adjacent counties within Western WA). This ensures that all comparisons are performed on the same number of pairs of counties. On each subsampled dataset, we compute the p-value from a Wilcoxon test evaluating differences between the relative risk of observing identical sequences in adjacent and non-adjacent counties. This is done for 1,000 subsampled datasets. Boxplots indicate the p-values obtained across these different subsampling iterations (5%, 25%, 50%, 75% and 95% quantiles). We do a similar analysis at the ZCTA level.

medRxiv preprint doi: https://doi.org/10.1101/2024.05.24.24307811; this version posted May 25, 2024. The copyright holder for this preprint (which was not certified by peer review) is the author/funder, who has granted medRxiv a license to display the preprint in perpetuity.
This article is a US Government work. It is not subject to copyright under 17 USC 105 and is also made available for use under a CC0 license.

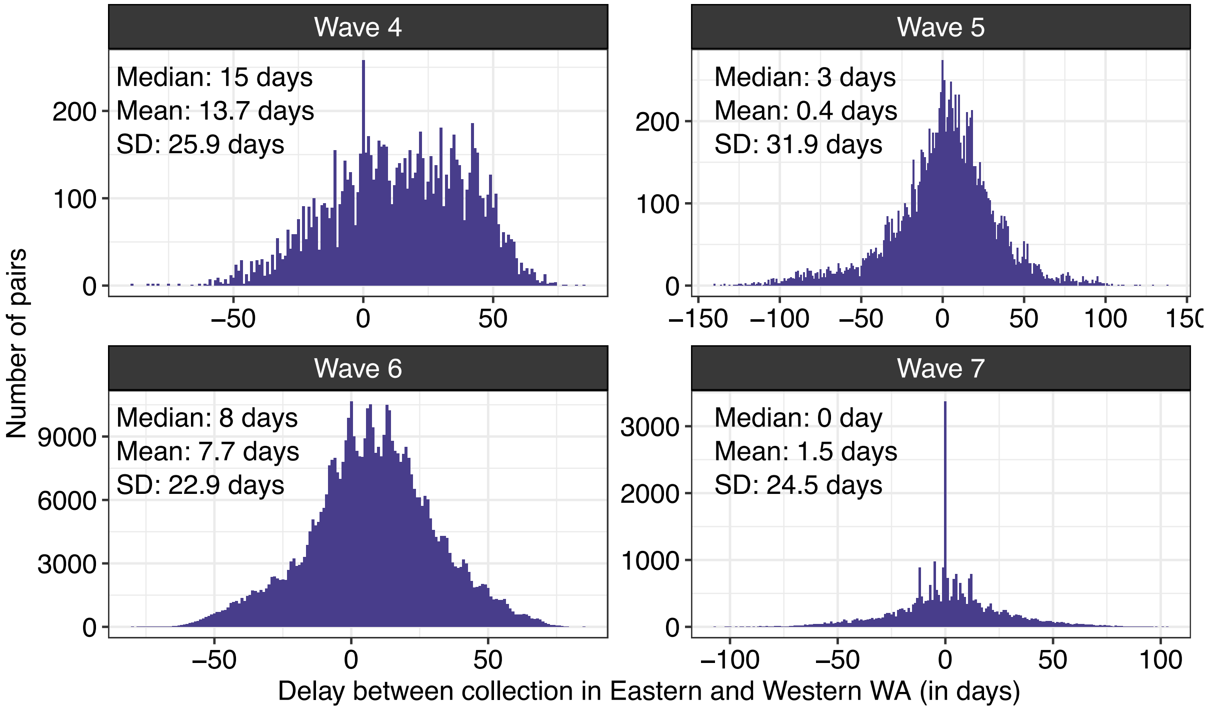

Figure S8. Distribution of the delay between sequence collection within pairs of identical sequences collected between Eastern and Western WA and across epidemic waves.

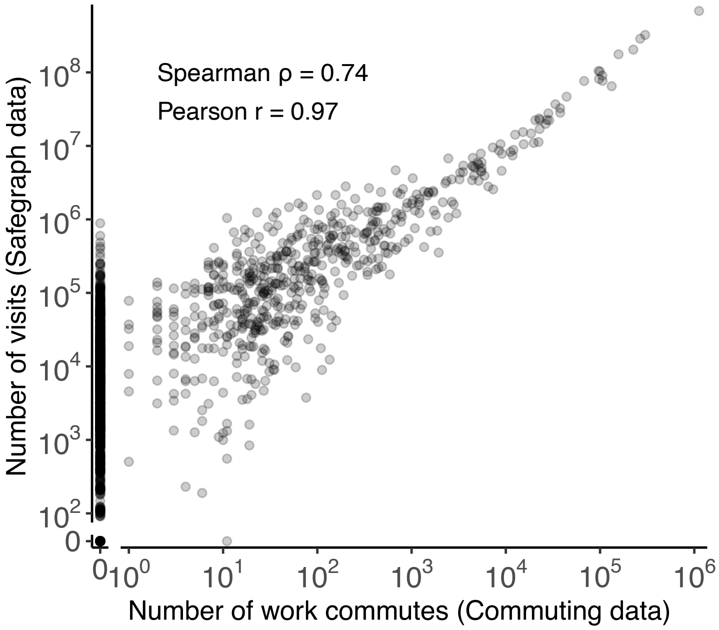

Figure S9. Comparison between the number of directed commuting flows and the number of directed visits between two counties. The number of work commutes is extracted from [13]. The number of visits is estimated using Safegraph *Weekly patterns* mobility data. The comparison is done by matching the origin county in the mobile phone data to the residence county in the workflow data and the destination county in the mobile phone data to the workplace county in the workflow data.

medRxiv preprint doi: https://doi.org/10.1101/2024.05.24.24307811; this version posted May 25, 2024. The copyright holder for this preprint (which was not certified by peer review) is the author/funder, who has granted medRxiv a license to display the preprint in perpetuity.
This article is a US Government work. It is not subject to copyright under 17 USC 105 and is also made available for use under a CC0 license.

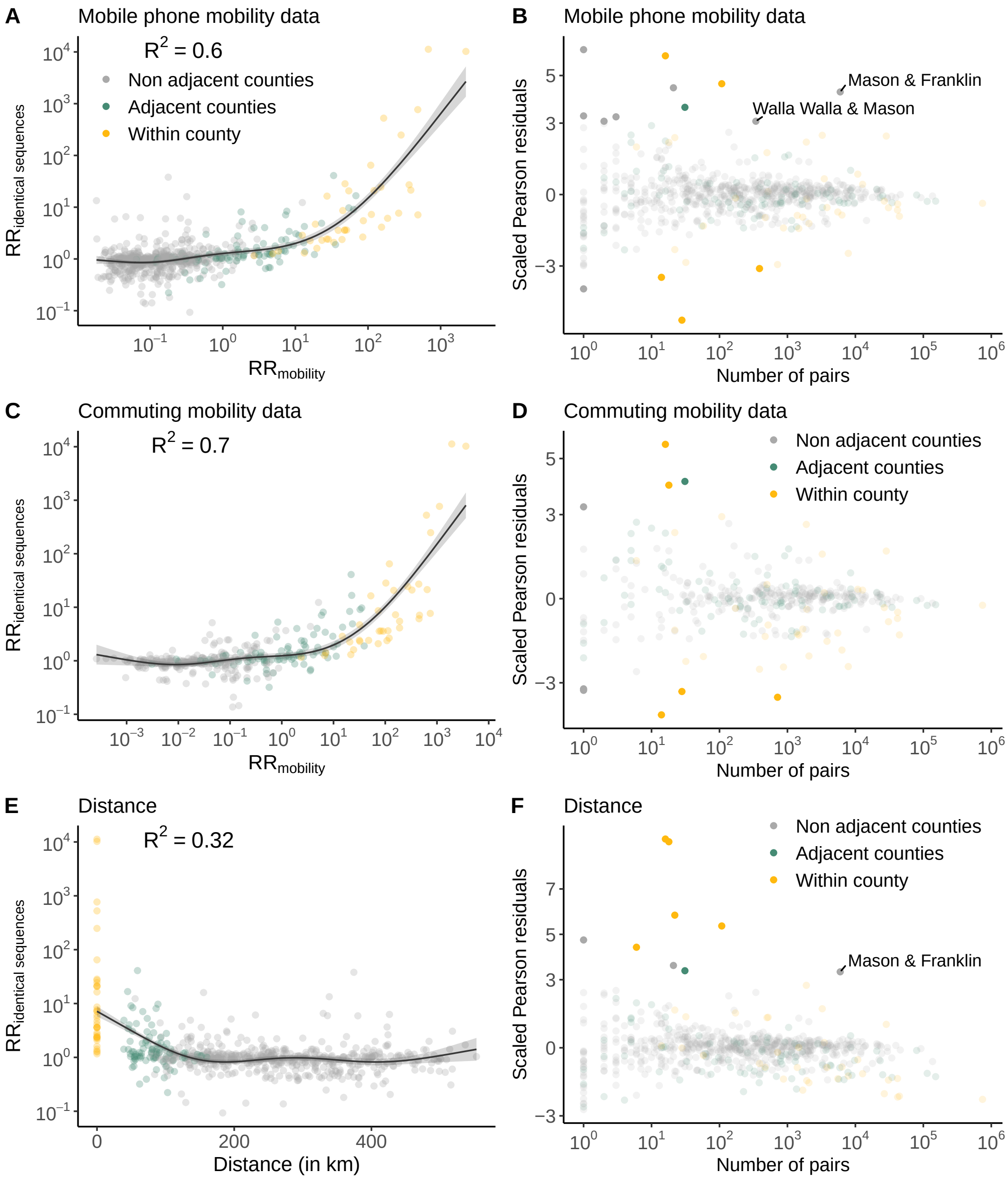

Figure S10. Comparison between the relative risk of observing identical sequences and the relative risk of movement at the county level. **A.** Relationship between the relative risk of observing identical sequences in two counties and the relative risk of movement between these counties as obtained from mobile phone mobility data. **B.** Scaled Pearson residuals of the GAM plotted in A as a function of the number of pairs of identical sequences observed in pairs of counties. **C.** Relationship between the relative risk of observing identical sequences in two counties and the relative risk of movement between these counties as obtained from workflow mobility data. **D.** Scaled Pearson residuals of the GAM plotted in C as a function of the number of pairs of identical sequences observed in pairs of counties. **E.** Relationship between the relative risk of observing identical sequences in two counties and the Euclidean distance between counties centroids. **F.** Scaled Pearson residuals of the GAM plotted in E as a function of the number of pairs of identical sequences observed in pairs of counties. In B, D and F, we label pairs of non-adjacent counties sharing at least 100 pairs of identical sequences and for which the absolute value of the Scaled Pearson residual is greater than 3. The trend lines correspond to predicted relative risk of observing identical sequences in two regions from each GAM. R^2 indicate the variance explained by each GAM.

medRxiv preprint doi: https://doi.org/10.1101/2024.05.24.24307811; this version posted May 25, 2024. The copyright holder for this preprint
(which was not certified by peer review) is the author/funder, who has granted medRxiv a license to display the preprint in perpetuity.
This article is a US Government work. It is not subject to copyright under 17 USC 105 and is also made available
for use under a CC0 license.

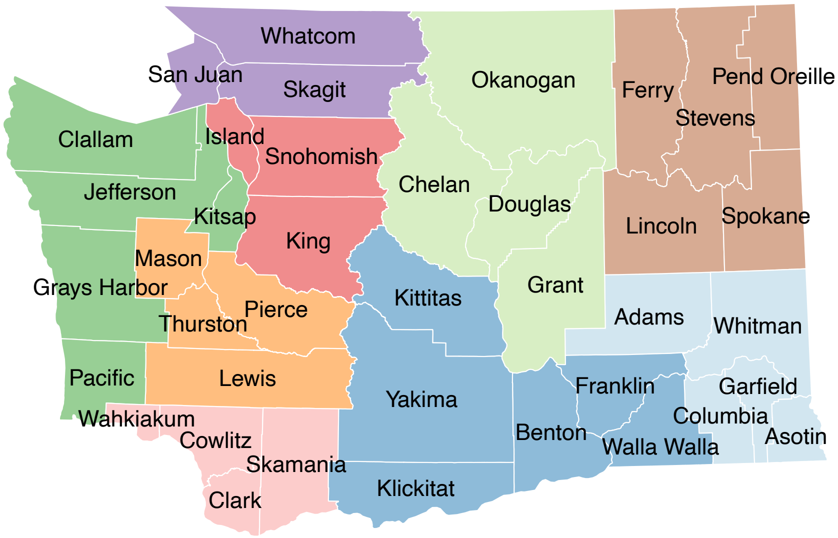

Figure S11. Geographical regions used to aggregate counties. Dark green: Peninsula/Coastal. Orange: South Puget Sound. Purple: Northwest. Red: North Puget Sound. Pink: Southwest. Dark blue: South Central. Light blue: Southeast.Brown: Northeast. Light green: North Central.

medRxiv preprint doi: https://doi.org/10.1101/2024.05.24.24307811; this version posted May 25, 2024. The copyright holder for this preprint (which was not certified by peer review) is the author/funder, who has granted medRxiv a license to display the preprint in perpetuity. This article is a US Government work. It is not subject to copyright under 17 USC 105 and is also made available for use under a CC0 license.

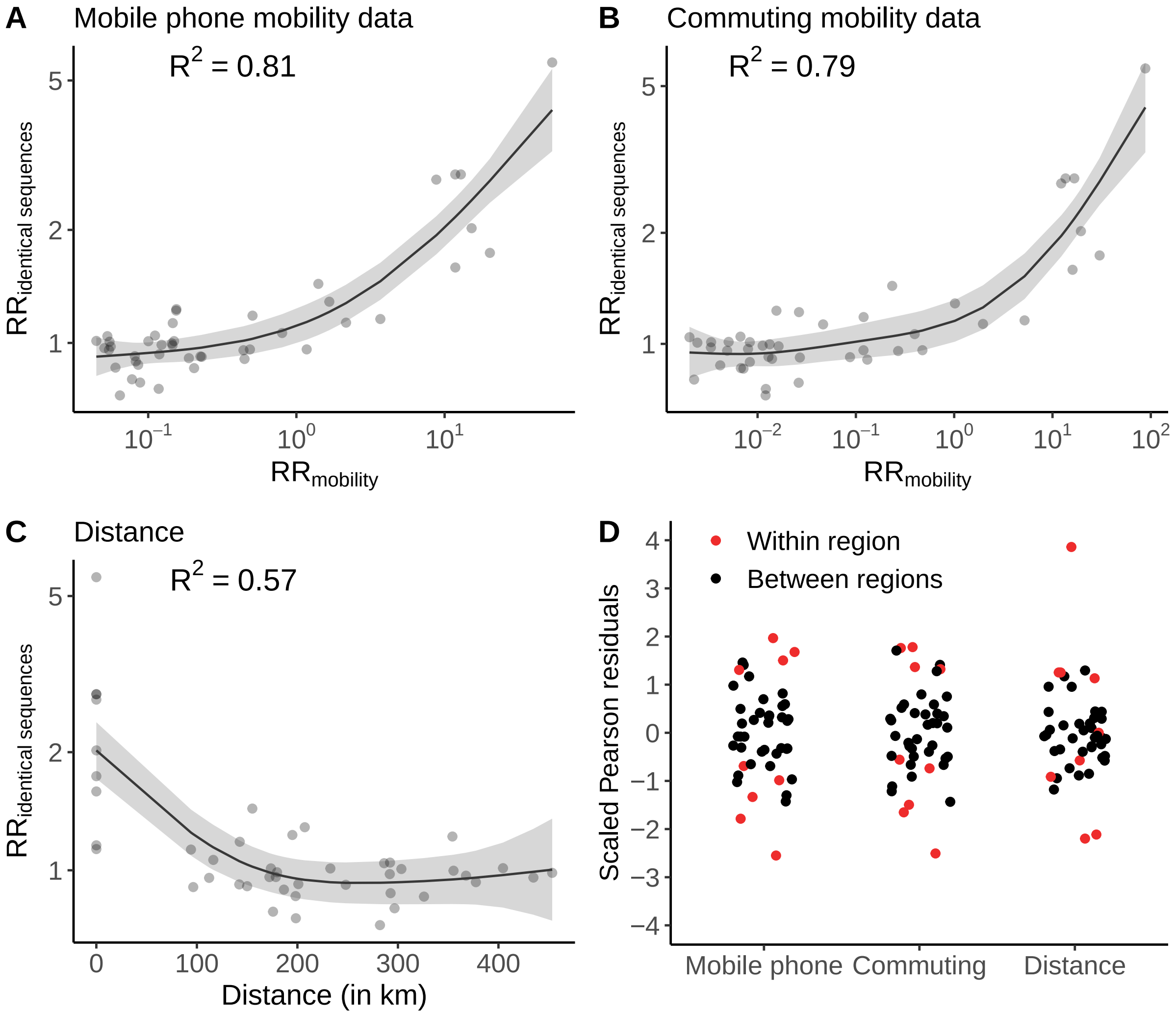

Figure S12. Comparison between the relative risk of observing identical sequences and the relative risk of movement at the region level. **A.** Relationship between the relative risk of observing identical sequences in two regions and the relative risk of movement between these regions as obtained from mobile phone mobility data. **B.** Relationship between the relative risk of observing identical sequences in two regions and the relative risk of movement between these regions as obtained from workflow mobility data. **C.** Relationship between the relative risk of observing identical sequences in two regions and the euclidean distance between region centroids. **D.** Scaled Pearson residuals of the GAM between the relative risk of observing identical sequences in two regions and (i) the relative risk of movement from commuting data, (ii) the relative risk of movement from mobile phone data and (iii) the geographic distance between regions' centroids. The trend lines correspond to predicted relative risk of observing identical sequences in two regions from each GAM. R^2 indicate the variance explained by each GAM.

medRxiv preprint doi: https://doi.org/10.1101/2024.05.24.24307811; this version posted May 25, 2024. The copyright holder for this preprint
(which was not certified by peer review) is the author/funder, who has granted medRxiv a license to display the preprint in perpetuity.
This article is a US Government work. It is not subject to copyright under 17 USC 105 and is also made available
for use under a CC0 license.

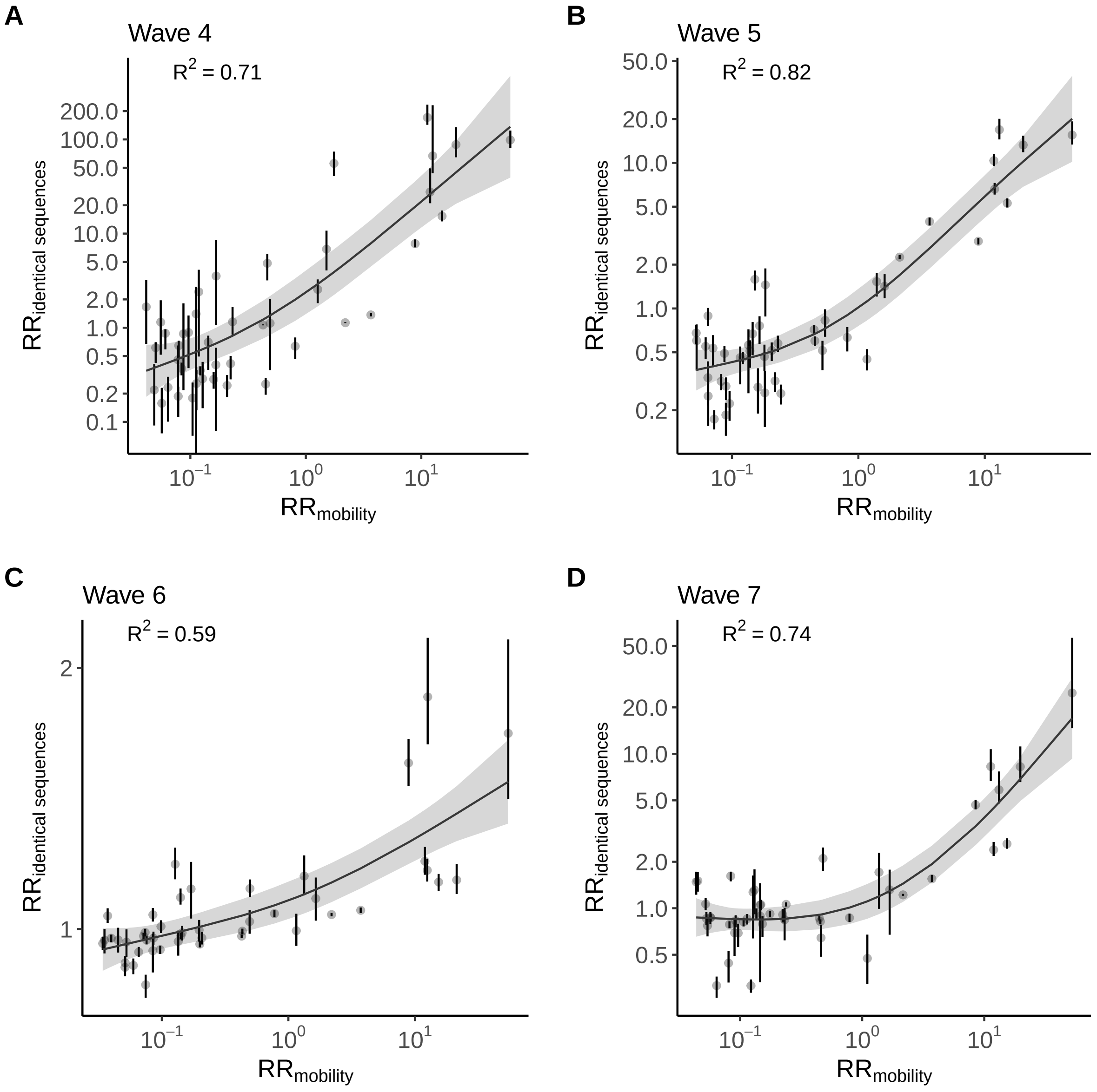

Figure S13. Relationship between the relative risk of observing identical sequences in two regions and the relative risk of movement between these regionsobtained from mobile phone mobility data across epidemic waves A. Wave 4. **B.** Wave 5. **C.** Wave 6. **D.** Wave 7. Vertical segments indicate 95% subsampling confidence intervals. The trend line correspond to predicted relative risk of observing identical sequences in two regions from a GAM. R^2 indicate the variance explained by each GAM.

medRxiv preprint doi: https://doi.org/10.1101/2024.05.24.24307811; this version posted May 25, 2024. The copyright holder for this preprint
(which was not certified by peer review) is the author/funder, who has granted medRxiv a license to display the preprint in perpetuity.
This article is a US Government work. It is not subject to copyright under 17 USC 105 and is also made available
for use under a CC0 license.

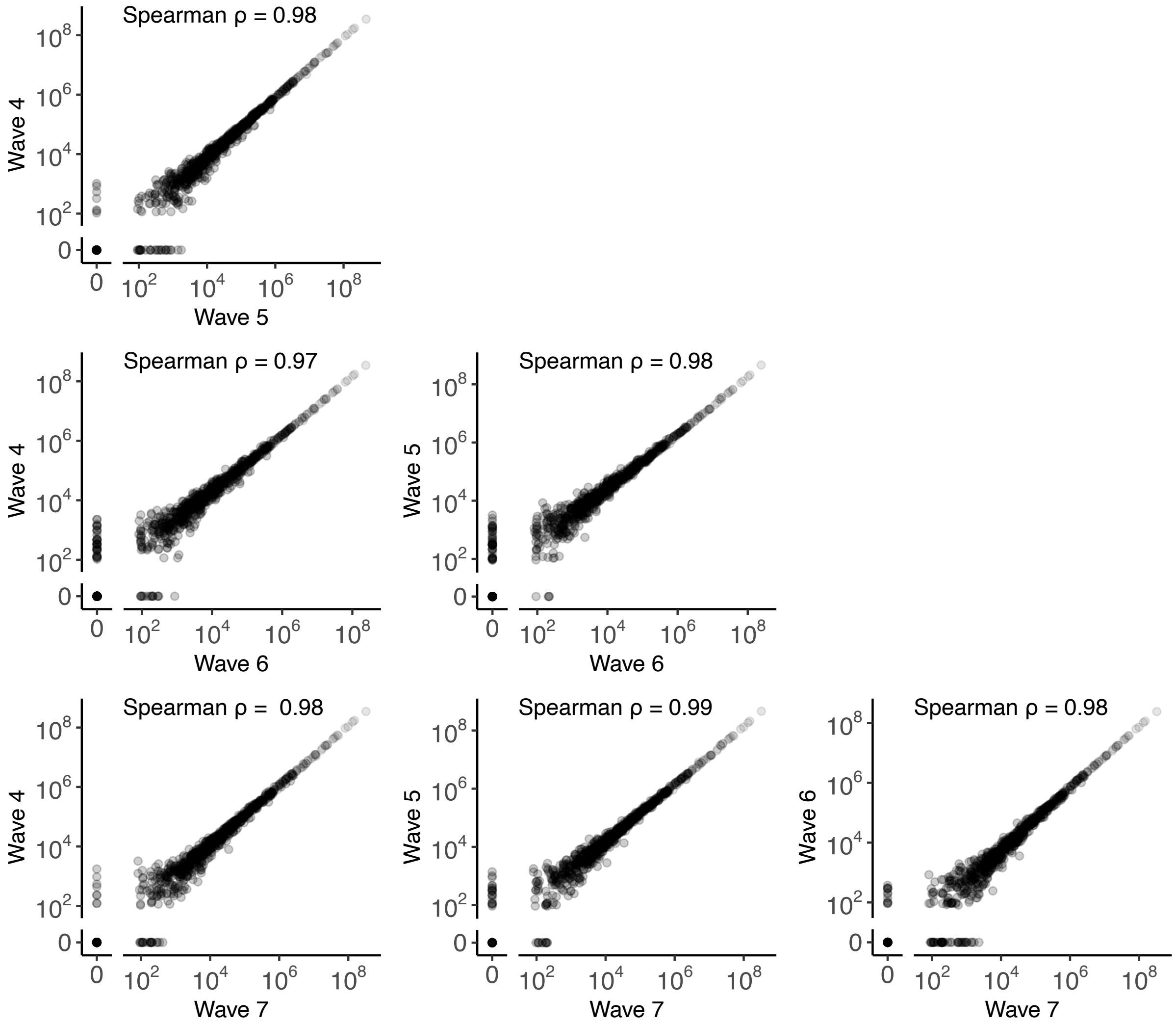

Figure S14. Comparison of the total number of visits between pairs of WA counties across the 4 epidemic waves during our study period. Points indicate the total number of visits between pairs of counties over the study periods labeled on the plot axes.

medRxiv preprint doi: https://doi.org/10.1101/2024.05.24.24307811; this version posted May 25, 2024. The copyright holder for this preprint (which was not certified by peer review) is the author/funder, who has granted medRxiv a license to display the preprint in perpetuity.
This article is a US Government work. It is not subject to copyright under 17 USC 105 and is also made available for use under a CC0 license.

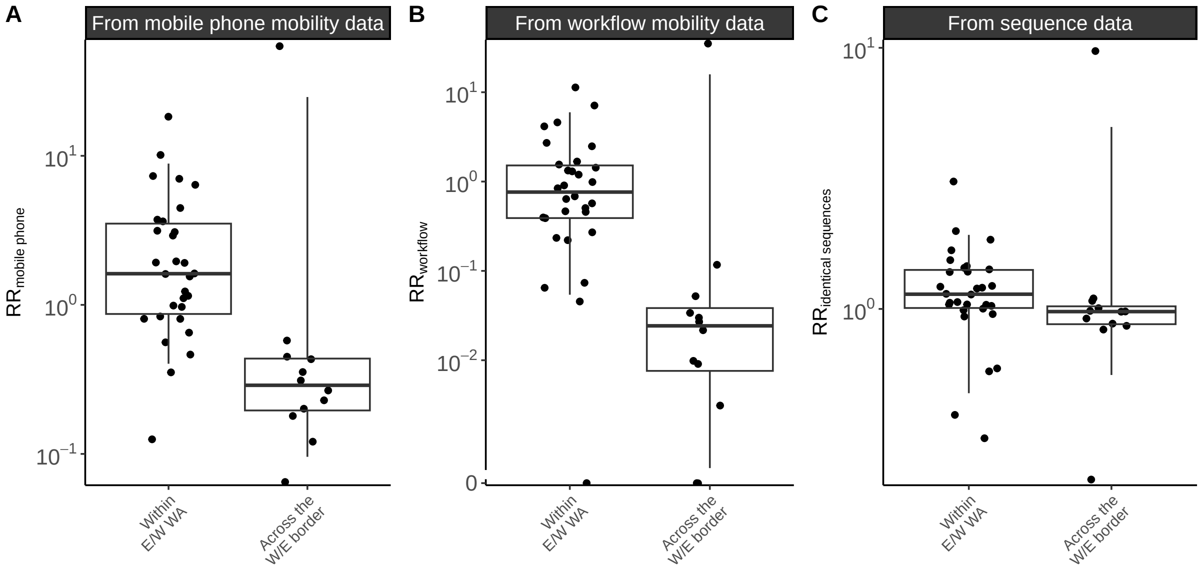

Figure S15. Comparison of connectivity metrics across the Eastern / Western WA border among counties located on the border. **A.** Relative risk of movement from mobile phone data across the border or within Eastern / Western WA (p-value for Wilcoxon rank sum test of $6.1 \cdot 10^{-5}$). **B.** Relative risk of movement from commuting data across the border or within Eastern / Western WA (p-value for Wilcoxon rank sum test of $1.6 \cdot 10^{-4}$). **C.** Relative risk of observing identical sequences across the border or within Eastern / Western WA (p-value for Wilcoxon rank sum test of $2.5 \cdot 10^{-2}$). In this analysis, we only consider WA counties along the W/E border.

medRxiv preprint doi: https://doi.org/10.1101/2024.05.24.24307811; this version posted May 25, 2024. The copyright holder for this preprint (which was not certified by peer review) is the author/funder, who has granted medRxiv a license to display the preprint in perpetuity. This article is a US Government work. It is not subject to copyright under 17 USC 105 and is also made available for use under a CC0 license.

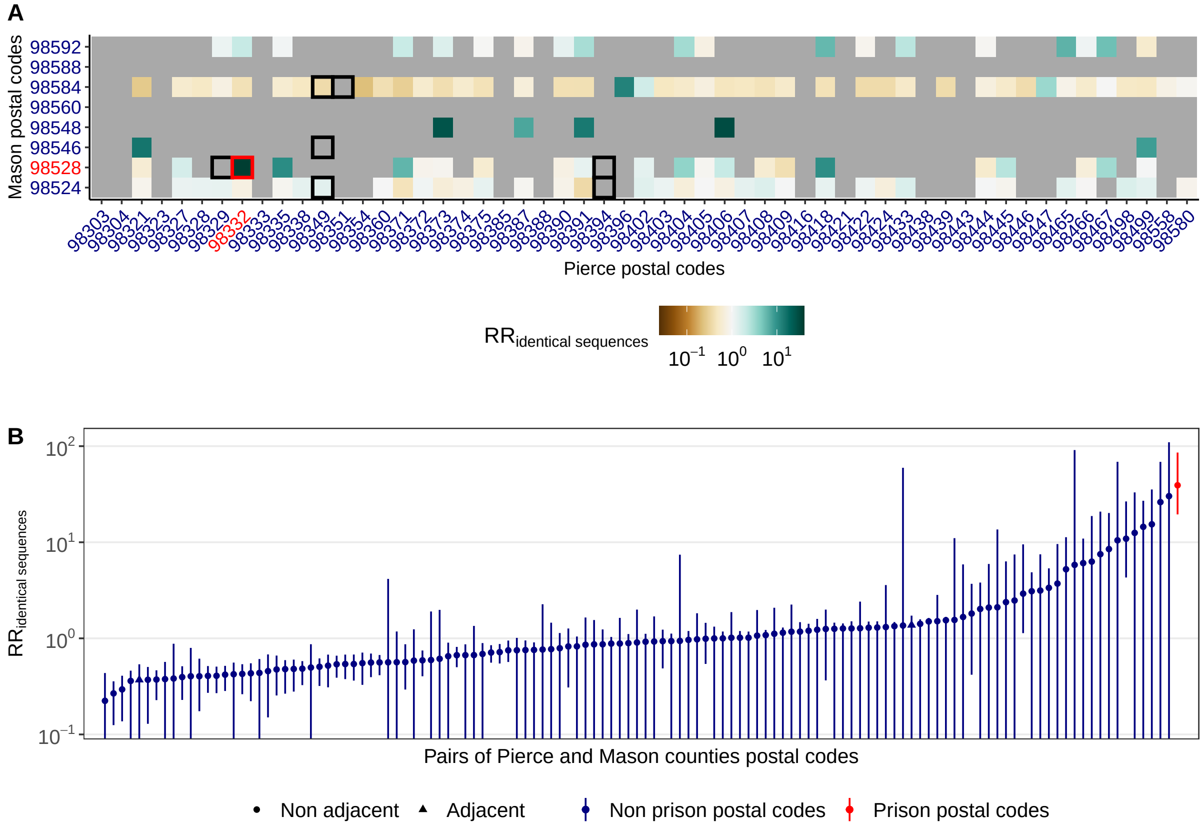

Figure S16. Patterns of occurrence of pairs of identical sequences between ZCTAs in Pierce and Mason counties, the two counties that are home of WA female prisons. A. Relative risk of observing identical sequences between ZCTAs in Mason and Pierce counties. Black squares indicate adjacent ZCTAs. ZCTAs in red correspond to postal codes that are the home of female prisons. **B.** Relative risk of observing identical sequences between Mason and Pierce counties ZCTAs ordered by increasing values. Vertical segments correspond to 95% subsampling confidence intervals.

medRxiv preprint doi: https://doi.org/10.1101/2024.05.24.24307811; this version posted May 25, 2024. The copyright holder for this preprint (which was not certified by peer review) is the author/funder, who has granted medRxiv a license to display the preprint in perpetuity.
This article is a US Government work. It is not subject to copyright under 17 USC 105 and is also made available for use under a CC0 license.

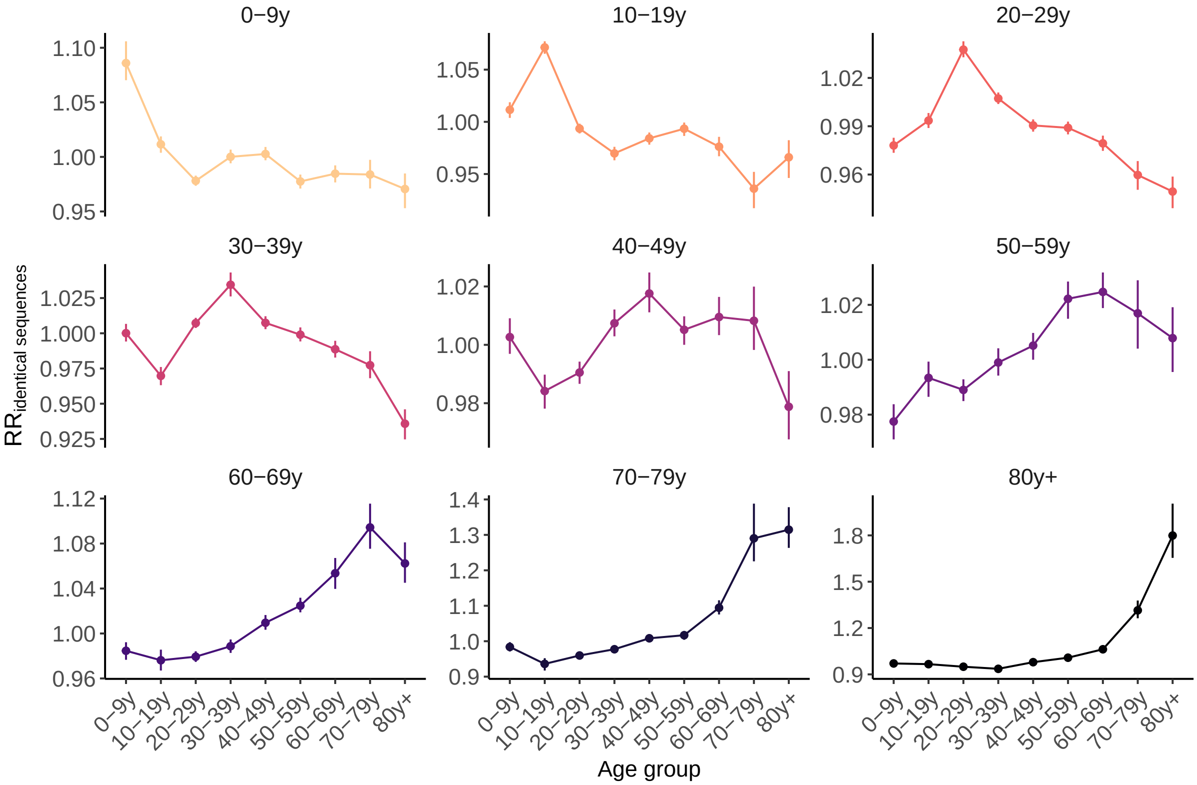

Figure S17. Relative risk for pairs of identical sequences of being observed between two age groups. Vertical segments correspond to 95% confidence intervals obtained through subsampling.

medRxiv preprint doi: https://doi.org/10.1101/2024.05.24.24307811; this version posted May 25, 2024. The copyright holder for this preprint (which was not certified by peer review) is the author/funder, who has granted medRxiv a license to display the preprint in perpetuity. This article is a US Government work. It is not subject to copyright under 17 USC 105 and is also made available for use under a CC0 license.

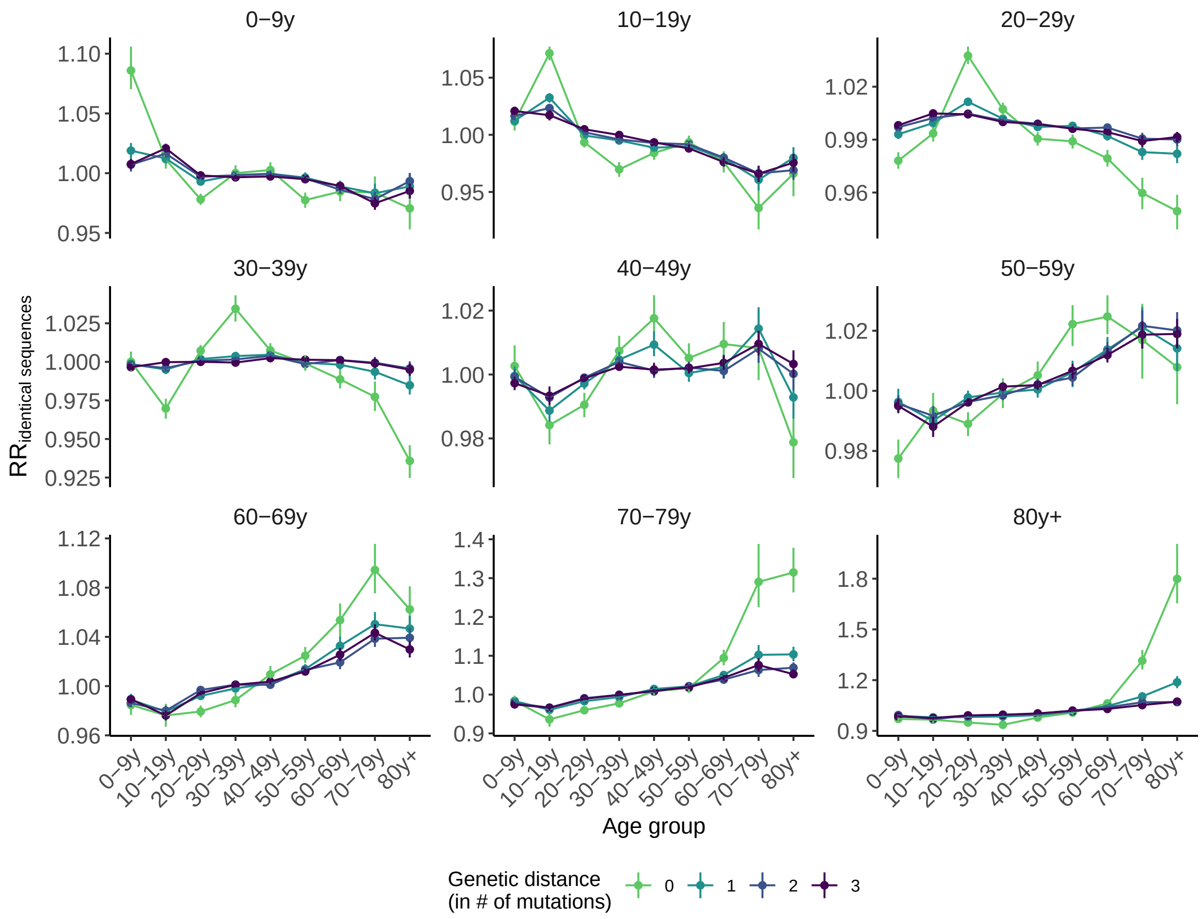

Figure S18. Relative risk for pairs sequences of being observed between two age groups depending on their genetic distance. Vertical segments correspond to 95% confidence intervals obtained through subsampling.

medRxiv preprint doi: https://doi.org/10.1101/2024.05.24.24307811; this version posted May 25, 2024. The copyright holder for this preprint (which was not certified by peer review) is the author/funder, who has granted medRxiv a license to display the preprint in perpetuity.
This article is a US Government work. It is not subject to copyright under 17 USC 105 and is also made available for use under a CC0 license.

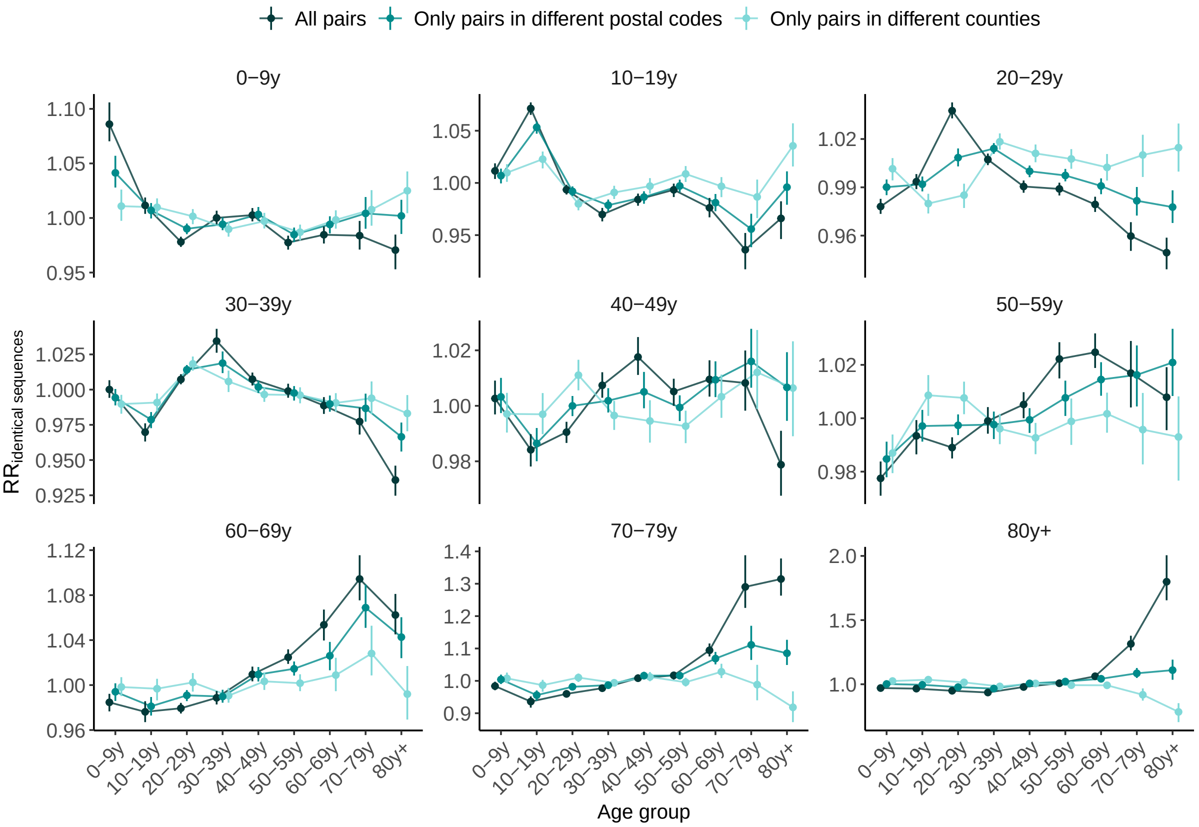

Figure S19. Impact of the spatial scale on the relative risk for pairs sequences of being observed between two age groups. Vertical segments correspond to 95% confidence intervals obtained through subsampling.

medRxiv preprint doi: https://doi.org/10.1101/2024.05.24.24307811; this version posted May 25, 2024. The copyright holder for this preprint (which was not certified by peer review) is the author/funder, who has granted medRxiv a license to display the preprint in perpetuity.
This article is a US Government work. It is not subject to copyright under 17 USC 105 and is also made available for use under a CC0 license.

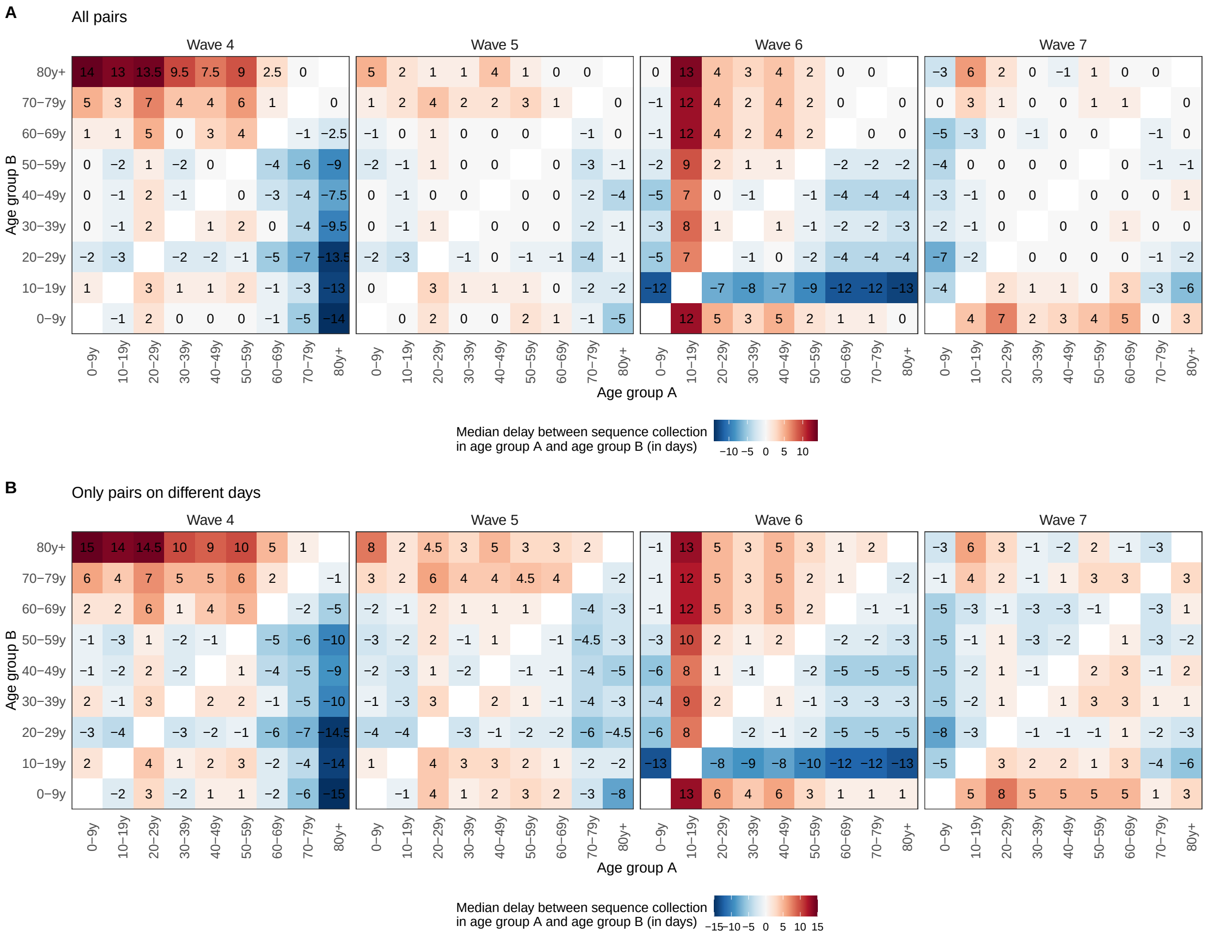

Figure S20. Median delay between the dates of sequence collection within pairs of identical sequences A. considering all pairs of identical sequences collected in two age groups and **B.** considering only pairs of identical sequences collected on different days in two age groups.

medRxiv preprint doi: https://doi.org/10.1101/2024.05.24.24307811; this version posted May 25, 2024. The copyright holder for this preprint (which was not certified by peer review) is the author/funder, who has granted medRxiv a license to display the preprint in perpetuity.
This article is a US Government work. It is not subject to copyright under 17 USC 105 and is also made available for use under a CC0 license.

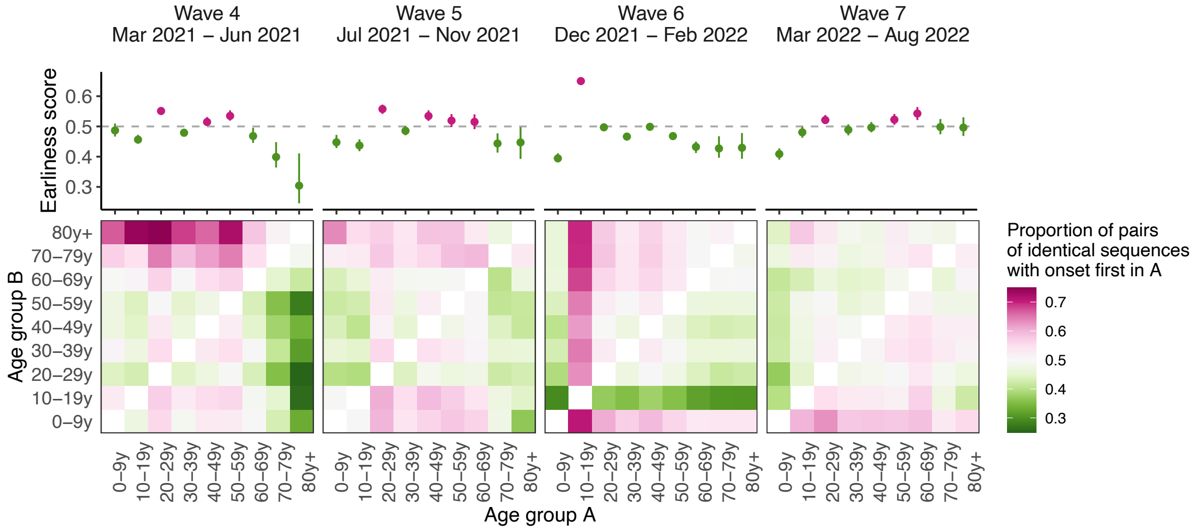

Figure S21. Sensitivity analysis on the timing of pairs identical sequences between age groups using symptom onset dates Median proportion of pairs of identical sequences with onset dates in age groups A before age group B across different epidemic waves from 1,000 imputed datasets (heatmaps). The dots plots depict the median earliness scores of age group *A* across 1,000 imputed datasets for the different epidemic waves. Vertical segments indicate uncertainty range around earliness score (see Methods).

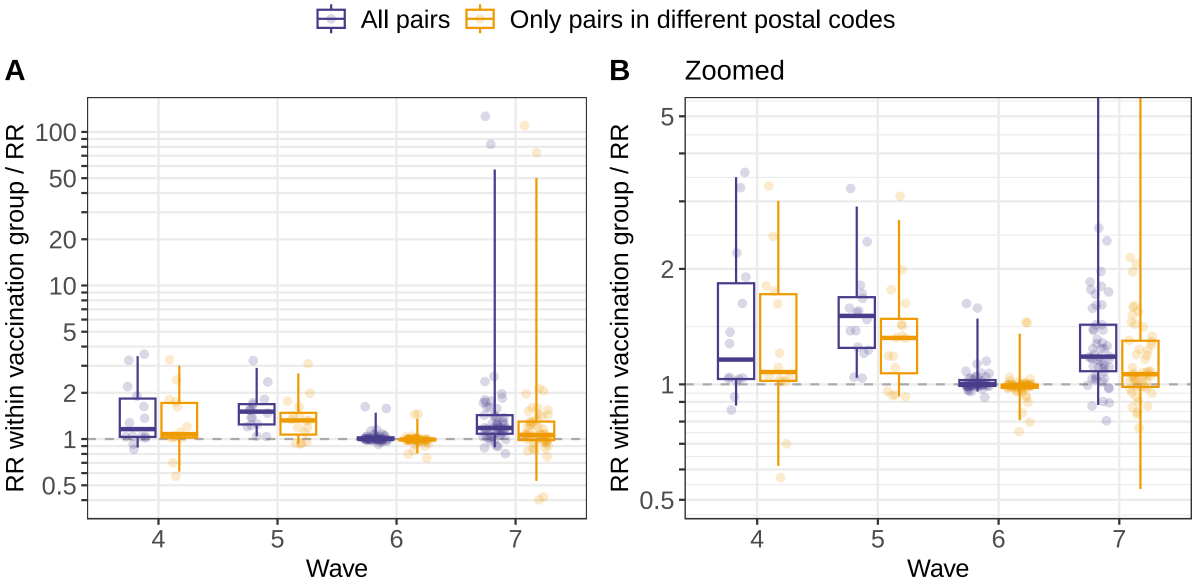

Figure S22. Ratio between the relative risk of observing identical sequences within a given vaccination group (denoted V_1) and between two vaccination groups (denoted V_1 and V_2). Values above 1 indicate that pairs of identical sequences tend to be enriched in pairs observed within the same vaccination group. The analysis is restricted to pairs observed within the same age group. Each point correspond to the ratio computed for a given pair of vaccination status (V_1, V_2) and age group. Boxplots indicate the 2.5%, 25%, 50%, 75% and 97.5% percentiles.

medRxiv preprint doi: https://doi.org/10.1101/2024.05.24.24307811; this version posted May 25, 2024. The copyright holder for this preprint (which was not certified by peer review) is the author/funder, who has granted medRxiv a license to display the preprint in perpetuity. This article is a US Government work. It is not subject to copyright under 17 USC 105 and is also made available for use under a CC0 license.

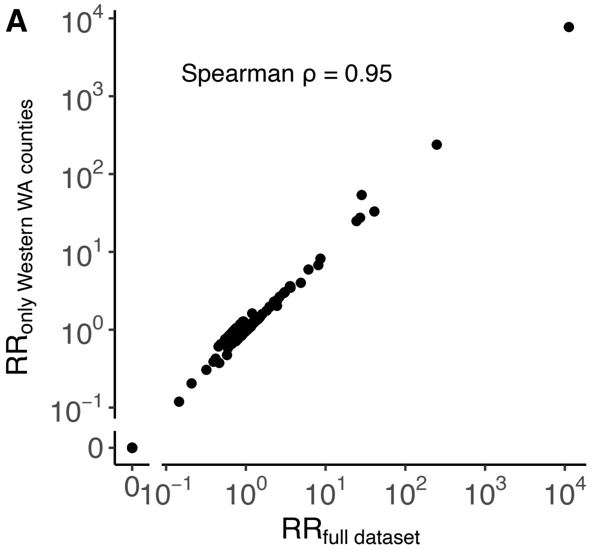

Figure S23. Impact of non-sampled locations on the computation of the RR. A. Comparison between the relative risk of observing identical sequences between Western WA counties using only sequence in Western WA counties or the entire sequence dataset. **B.** Comparison between the relative risk of observing identical sequences between Eastern WA counties using only sequence in Eastern WA counties or the entire sequence dataset.

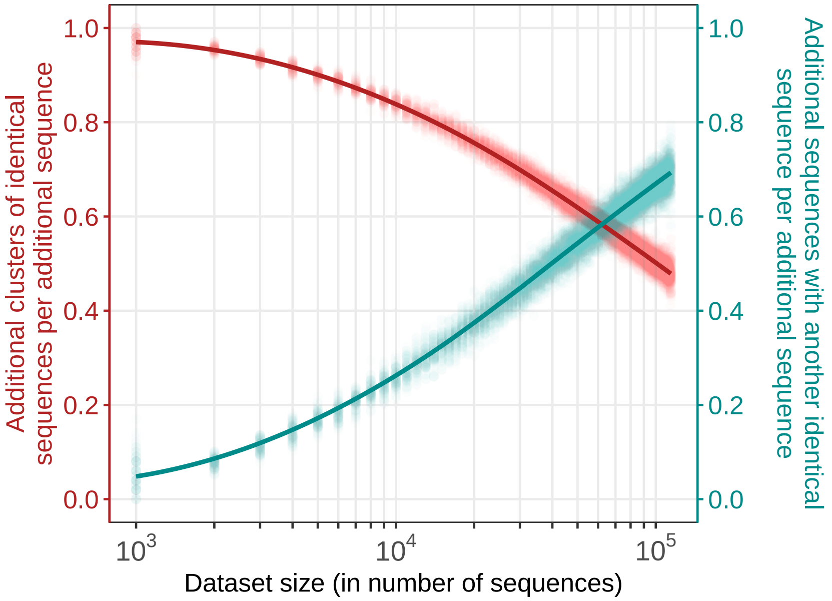

Figure S24. Impact of dataset size on the number of clusters of identical sequences and the number of sequences with another identical sequence in the dataset. We generate this figure by considering sequence datasets of increasing sizes, ranging between 10^2 and the 114,298 (the size of our WA dataset) with an increment of 10^2 between 10^2 and 10^3 and an increment of 10^3 above 10^3. We run 100 simulations where we first downsample 10^2 sequences from our full dataset and then incrementally include more sequences (drawn from the total remaining sequences not yet included). At every step, we compute the additional number of clusters of identical sequences per additional sequences (red) as well as the additional number of sequences with another identical sequence in the dataset per additional sequence (cyan). Points indicate the results from individual simulations and lines the LOESS curves.

medRxiv preprint doi: https://doi.org/10.1101/2024.05.24.24307811; this version posted May 25, 2024. The copyright holder for this preprint (which was not certified by peer review) is the author/funder, who has granted medRxiv a license to display the preprint in perpetuity.
This article is a US Government work. It is not subject to copyright under 17 USC 105 and is also made available for use under a CC0 license.

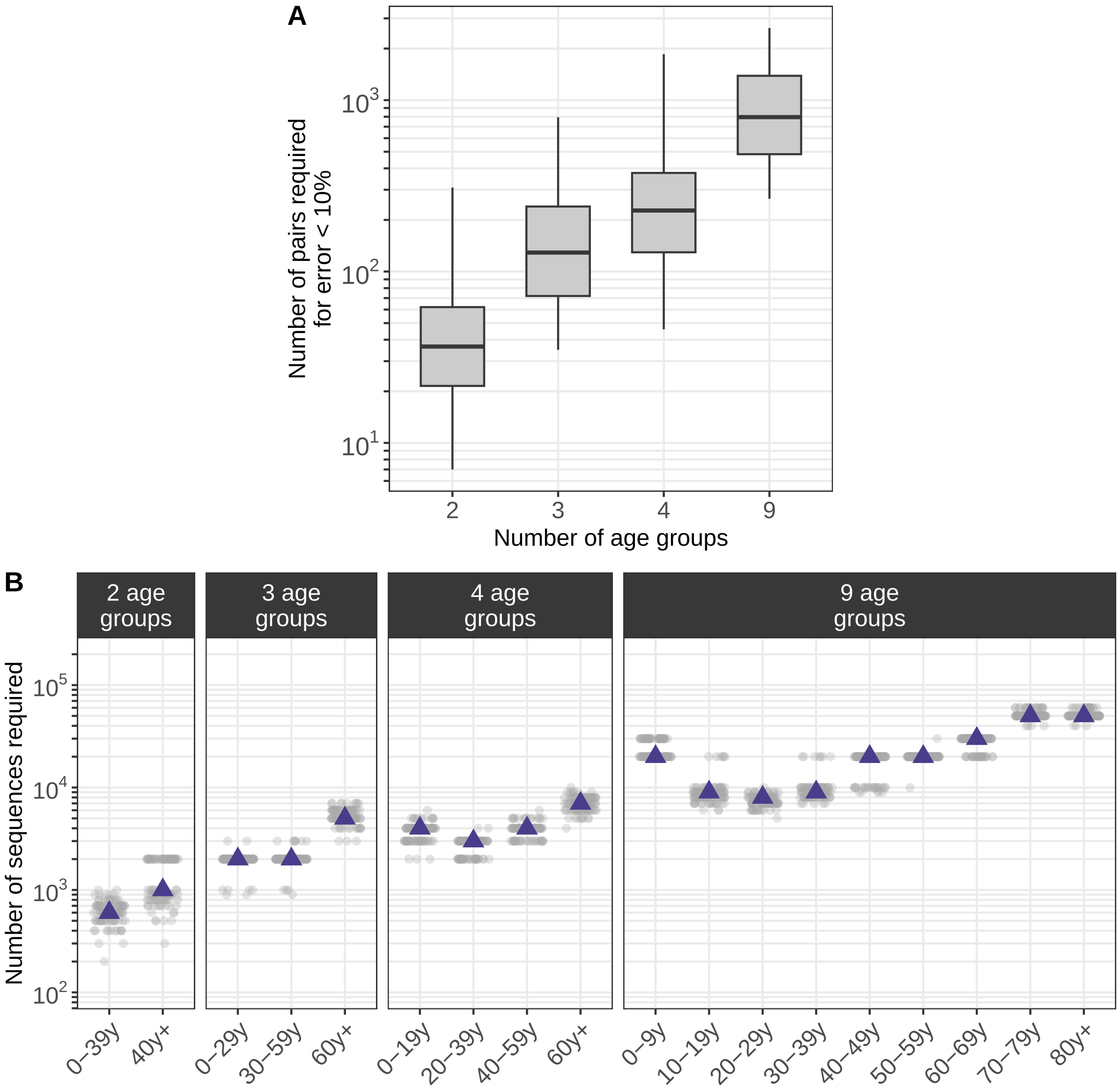

Figure S25. Impact of the number of groups included in the analysis on the dataset size required for the error relative risk of observing identical sequences to be lower than 10%. A. Number of pairs of identical sequences required for the error relative risk of observing identical sequences to be lower than 10%. Boxplots indicate the 2.5%, 25%, 50%, 75% and 97.5% percentiles. See Methods for a description of the downsampling strategy. **B.** Number of sequences required for the number of pairs of identical sequences observed within the age group on the x-axis to reach the median depicted in A. Each point corresponds to a subsampled dataset. Purple triangles indicate the median.

medRxiv preprint doi: https://doi.org/10.1101/2024.05.24.24307811; this version posted May 25, 2024. The copyright holder for this preprint
(which was not certified by peer review) is the author/funder, who has granted medRxiv a license to display the preprint in perpetuity.
This article is a US Government work. It is not subject to copyright under 17 USC 105 and is also made available
for use under a CC0 license.

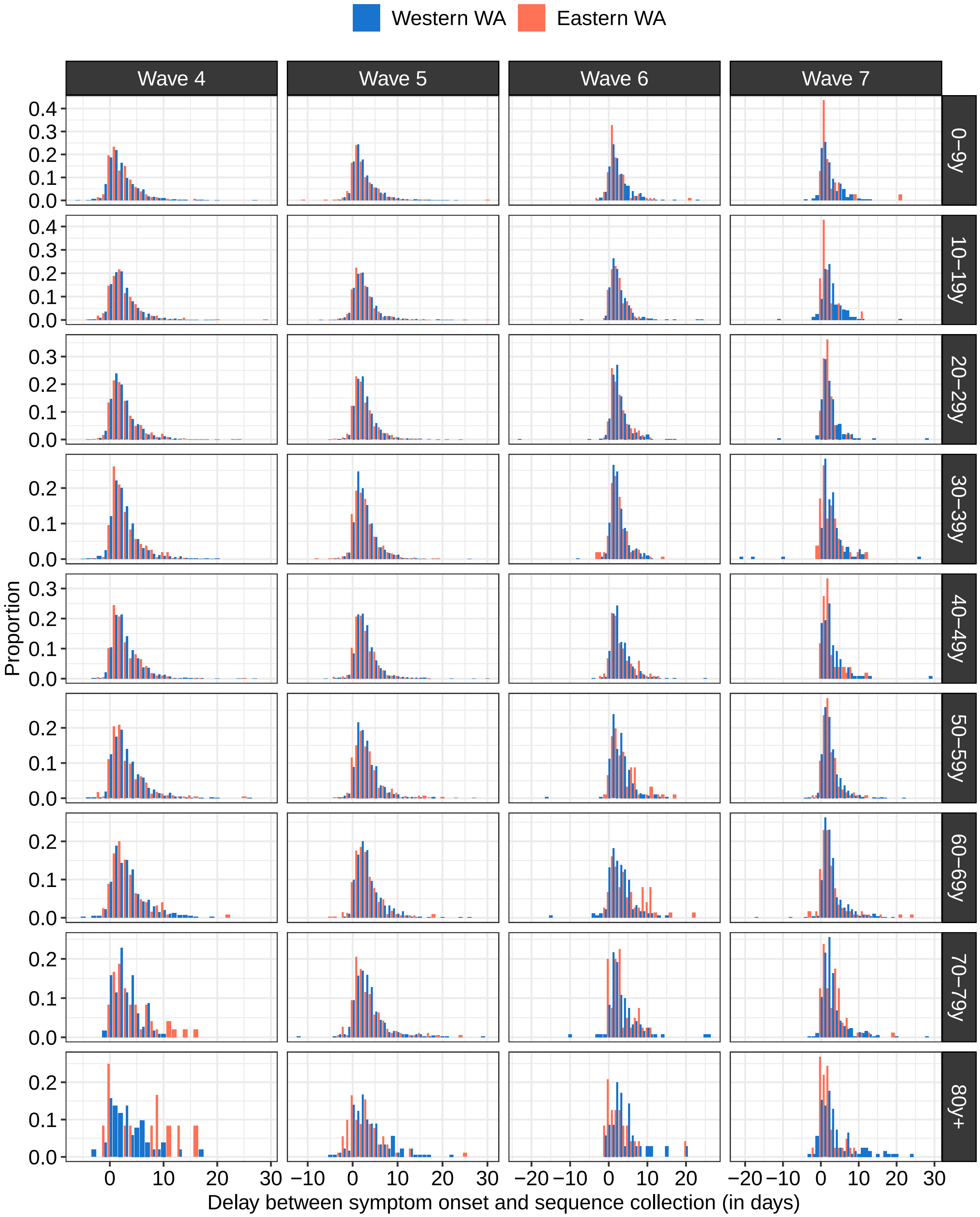

Figure S26. Empirical distribution of the delay between symptom onset and sequence collection by age (rows), period (columns) and geographic region (colours).

medRxiv preprint doi: https://doi.org/10.1101/2024.05.24.24307811; this version posted May 25, 2024. The copyright holder for this preprint (which was not certified by peer review) is the author/funder, who has granted medRxiv a license to display the preprint in perpetuity. This article is a US Government work. It is not subject to copyright under 17 USC 105 and is also made available for use under a CC0 license.

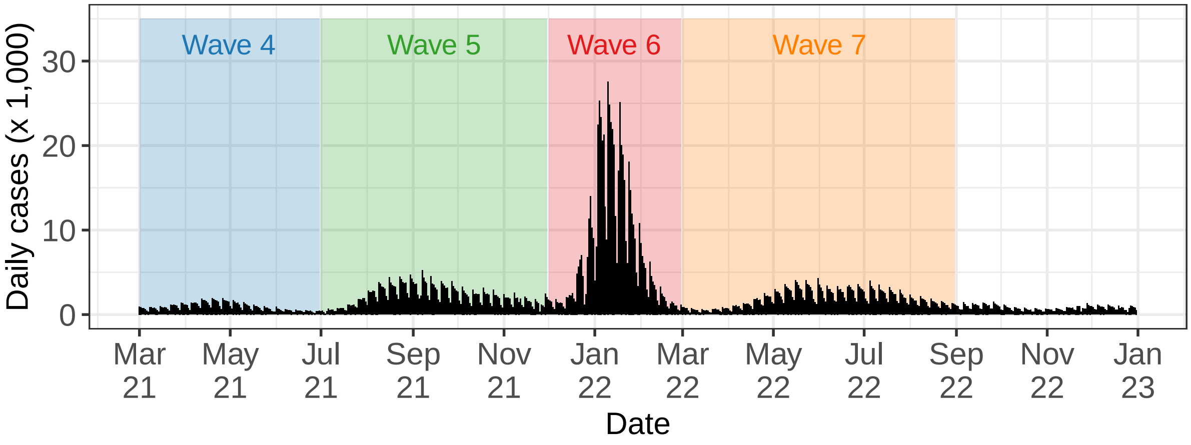

Figure S27. Time-series of COVID-19 cases in WA over the study periods. Shaded rectangles indicate the periods used to define the successive epidemic waves.

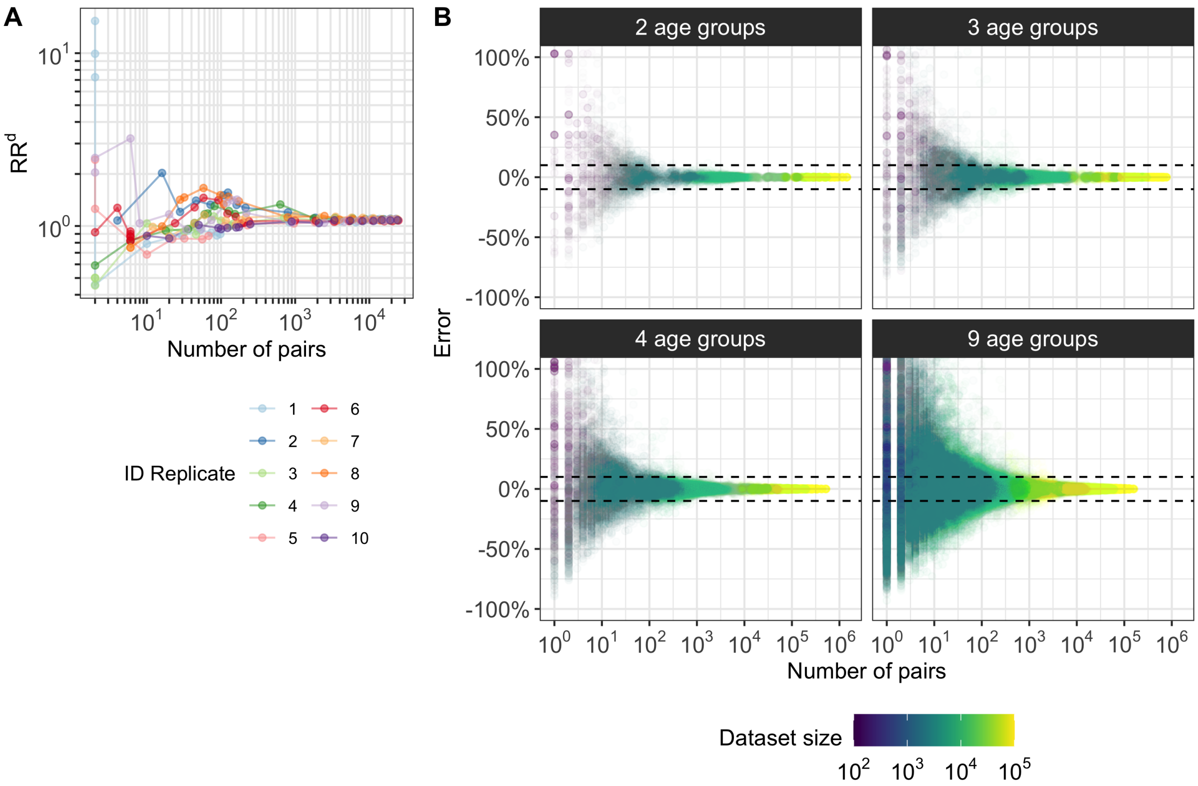

Figure S28. Illustration of the downsampling strategy used to quantify the amount of data required to compute relative risks. A. Relative risk RR^d of identical sequences being shared between the 0-9y computed on 10 downsampled datasets as a function of the number of pairs of identical sequences shared between the 0-9y. **B.** Error ϵ on the relative risk of observing identical sequences in downsampled datasets as a function of the number of pairs of identical sequences present for a pair of age groups in the downsampled datasets.

medRxiv preprint doi: https://doi.org/10.1101/2024.05.24.24307811; this version posted May 25, 2024. The copyright holder for this preprint (which was not certified by peer review) is the author/funder, who has granted medRxiv a license to display the preprint in perpetuity.
This article is a US Government work. It is not subject to copyright under 17 USC 105 and is also made available for use under a CC0 license.

Method	Average sequencing probability	Replicate	Sample size (unbiased)	Sample size (biased)	Corr. with sim (unbiased)	Corr. with sim (biased)	Corr. biased / unbiased
**DTA** Fixing tree	0.43 %	1	1745	1744	0.54	-0.22	0.10
**DTA** Fixing tree	0.43 %	2	1714	1805	0.60	0.19	0.39
**DTA** Fixing tree	2.16 %	1	8723	8709	0.61	0.39	0.84
**DTA** Fixing tree	2.16 %	2	8551	8675	0.77	0.25	0.76
**DTA** Inferring tree	0.43 %	1	1745	1744	0.10	0.15	-0.13
**RR**	2.16 %	1	8723	8709	0.94	0.80	0.79
**RR**	2.16 %	2	8551	8675	0.80	0.75	0.86
**RR**	8.66 %	1	34338	35304	0.91	0.74	0.77
**RR**	8.66 %	2	34736	35123	0.93	0.84	0.79

Table S1. Performance of Discrete Trait Analysis (DTA) and our relative risk metric (RR) in quantifying migration patterns. The sample sizes correspond to the number of sequences on which the inference is performed. All correlation coefficients reported are Spearman rank correlation coefficients. In the DTA analysis, we report the correlation between estimated and true migration rates (both for the biased and unbiased sequencing scenarios) and the correlation between the migration rates estimated on the biased and unbiased datasets. In the RR analysis, we report the correlation between the RR and the migration probability between demes (both for the biased and unbiased sequencing scenarios) as well as the correlation between the RR estimated on the biased and unbiased datasets.

Region	Adjacency status compared	p-value (Wilcoxon test)	p-value (Wilcoxon test) without 0
East-East	Within ZCTA & Adjacent ZCTAs	$5 \cdot 10^{-10}$	$3 \cdot 10^{-12}$
	Adjacent ZCTAs & Non-adjacent ZCTAs	$< 10^{-16}$	$< 10^{-16}$
East-West	Adjacent ZCTAs & Non-adjacent ZCTAs	0.73	0.39
West-West	Within ZCTA & Adjacent ZCTAs	$< 10^{-16}$	$< 10^{-16}$
	Adjacent ZCTAs & Non-adjacent ZCTAs	$< 10^{-16}$	$< 10^{-16}$

Table S2. Comparison of the relative of risk of observing identical sequences at the ZCTA level by adjacency level. We report the p-values of Wilcoxon rank sum test using either all pairs of ZCTAs or only pairs of ZCTAs for which pairs of identical sequences are collected (column ”without 0”).

medRxiv preprint doi: https://doi.org/10.1101/2024.05.24.24307811; this version posted May 25, 2024. The copyright holder for this preprint (which was not certified by peer review) is the author/funder, who has granted medRxiv a license to display the preprint in perpetuity. This article is a US Government work. It is not subject to copyright under 17 USC 105 and is also made available for use under a CC0 license.

	At the county level	At the region level
Spearman correlation ρ		
Mobile phone mobility	35%	61%
Workflow mobility data	40%	59%
Geographic distance	-35%	-48%
Spearman correlation ρ (without 0)		
Mobile phone mobility	43%	61%
Workflow mobility data	56%	59%
Geographic distance	-36%	-48%
Variance explained (GAM)		
Mobile phone mobility	60%	81%
Workflow mobility data	70%	79%
Geographic distance	32%	57%

Table S3. Comparison between the relative risk of observing identical sequences between two geographic regions and the risk of movement between different geographies. We consider three data sources to inform the relative risk of movement between geographies: the relative risk for a visit to occur between two geographies (from mobile phone data), the relative risk for a work commute to occur between two geographies (from workflow data) and the geographic distance between geographies' centroids.

Facility name	County	Prison capacity	County population size	Ratio prison capacity / county population
Washington State Penitentiary	Walla Walla	2439	62584	$3.90 \cdot 10^{-2}$
Stafford Creek Corrections Center	Grays Harbor	1936	75636	$2.56 \cdot 10^{-2}$
Coyote Ridge Corrections Center	Franklin	2468	96749	$2.55 \cdot 10^{-2}$
Washington Corrections Center	Mason	1268	65726	$1.93 \cdot 10^{-2}$
Clallam Bay Corrections Center	Clallam	858	77155	$1.11 \cdot 10^{-2}$
Airway Heights Corrections Center	Spokane	2258	539339	$4.19 \cdot 10^{-3}$
Olympic Corrections Center	Clallam	272	77155	$3.53 \cdot 10^{-3}$
Monroe Correctional Complex	Snohomish	2400	827957	$2.90 \cdot 10^{-3}$
Cedar Creek Corrections Center	Thurston	480	294793	$1.63 \cdot 10^{-3}$
Larch Corrections Center	Clark	240	503311	$4.77 \cdot 10^{-4}$

Table S4. Characteristics of WA male prisons.
